# Antimalarial troponoids, puberulic acid and viticolins; divergent synthesis and structure-activity relationship studies

**DOI:** 10.1038/s41598-017-07718-3

**Published:** 2017-08-03

**Authors:** Goh Sennari, Ryo Saito, Tomoyasu Hirose, Masato Iwatsuki, Aki Ishiyama, Rei Hokari, Kazuhiko Otoguro, Satoshi Ōmura, Toshiaki Sunazuka

**Affiliations:** 1 Graduate School of Infection Control Sciences, Kitasato University, 5-9-1 Shirokane, Minato-ku, Tokyo 108-8641 Japan; 2 Kitasato Institute for Life Sciences, Kitasato University, 5-9-1 Shirokane, Minato-ku, Tokyo 108-8641 Japan

## Abstract

Divergent synthesis of antimalarial troponoids, including naturally occurring compounds, some of which were identified and isolated by our group, has been achieved utilizing the total synthetic route of puberulic acid. Structure-activity relationships of natural products and simple troponoids inspired us to explore more detailed properties of this class of compounds. Access to new derivatives was facilitated through intermediate compounds generated during the total synthesis of puberulic acid by a stepwise oxidation-aromatization sequence to provide 7-hydroxytropolones and bromination for conversion of the carboxylic acid moiety. The first total synthesis of viticolin A, as well as the synthesis of different methyl-substituted derivatives, has also been achieved. *In vitro* antimalarial activity and cytotoxicity of novel derivatives were evaluated and fundamental information to facilitate the discovery of more promising antimalarials was obtained.

## Introduction

As one of the most critical infectious diseases in the world, particularly in sub-Saharan Africa, malaria remains a serious global health problem. The World Health Organization (WHO) estimated that there were 212 million clinical cases of malaria and 429 000 deaths in 2015 and has been warning that it puts 3.2 billion people, about half of the world’s population, at risk^1^. In spite of various traditional antimalarial drugs such as quinine^2^, chloroquine^3^ and sulfadoxine/pyrimethamine^4^ having been developed for malaria, plus current artemisinin-based combination therapy (ACT)^5^, drug-resistant parasites and multi-drug resistance against ACT have been rapidly and continually emerging^6^. Development of antimalarial drugs with novel structures and new modes of action is, therefore, incessantly and urgently required.

**Figure 1:**
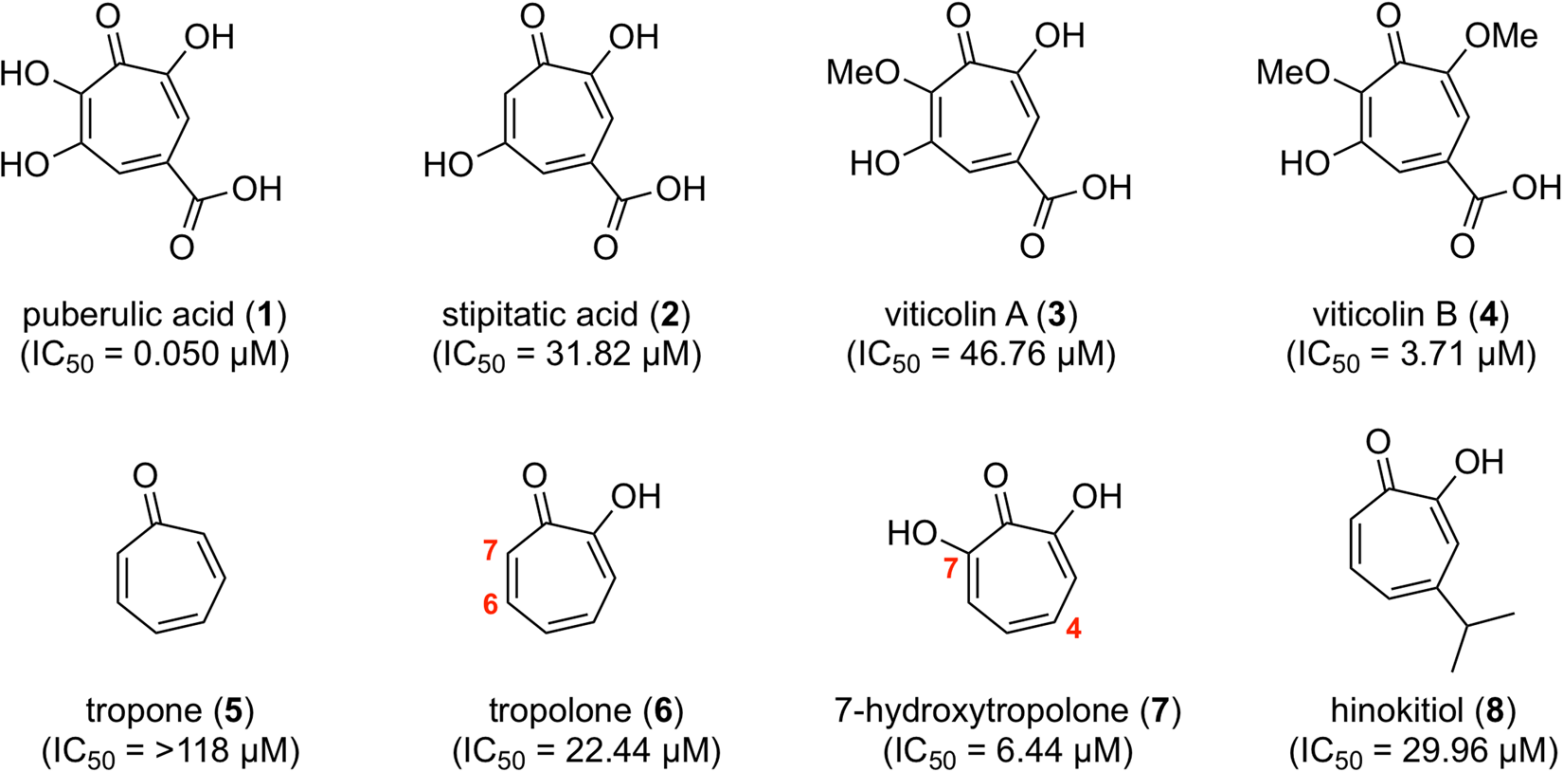
Antimalarial troponoids and their activity against *Plasmodium falciparum* K1 strain.

Puberulic acid (**1**)^7^, stipitatic acid (**2**)^8^ and viticolins A and B (**3**, **4**) as novel natural products have been isolated from a culture broth of *Penicillium viticola* ^9^ FKI-4410 through our screening system and found to have promising antimalarial activity (Fig. 1)^10, 11^. In these highly-oxygenated 7-membered aromatic compounds, **1** shows the most potent antimalarial activity *in vitro* against the *Plasmodium falciparum* K1 (chloroquine-resistant) parasite strain (IC_50_ = 0.050 µM), as well as *in vivo* efficacy with 69% inhibition for a dose of 2 mg/kg × 4 through subcutaneous (s.c.) administration in 4-day suppressive test using a *P. berghei*-infected mouse model^12^. However, **1** exhibits toxicity *in vivo*, four out of five mice dying by day 3, after a s.c. dose of 5 mg/kg × 2 (day 0 and 1). While structually simple compounds such as tropone (**5**), tropolone (**6**) and hinokitiol (**8**), and natural **2** and **3** showed weaker activity than that of **1**, 7-hydroxytropolone (**7**)^13^ was much more potent, exhibiting a >18-fold stronger IC_50_ value of 6.44 µM than that of **5**. This observation suggested that the presence of more than three contiguous oxygen atoms in a compound might significantly affect antimalarial activity. These results stimulated us to undertake a structure-activity relationship (SAR) study based on the establishment of a total synthetic route, aiming to create new antimalarial candidates which retained potency but which were non-toxic. Furthermore, we expected that these compounds’ properties, especially low molecular weight and simple planar structures, could be invaluable for antimalarial drug leads with respect to ease of supply^14^, enabled by efficient and practical synthesis. Herein, we report the divergent synthesis of several related troponoids, including natural products, via utilization of the established total synthetic route of **1** ^15^, and biological evaluation of their *in vitro* antimalarial activity and cytotoxicity.

## Results and Discussion

### Synthetic strategy

**Figure 2:**
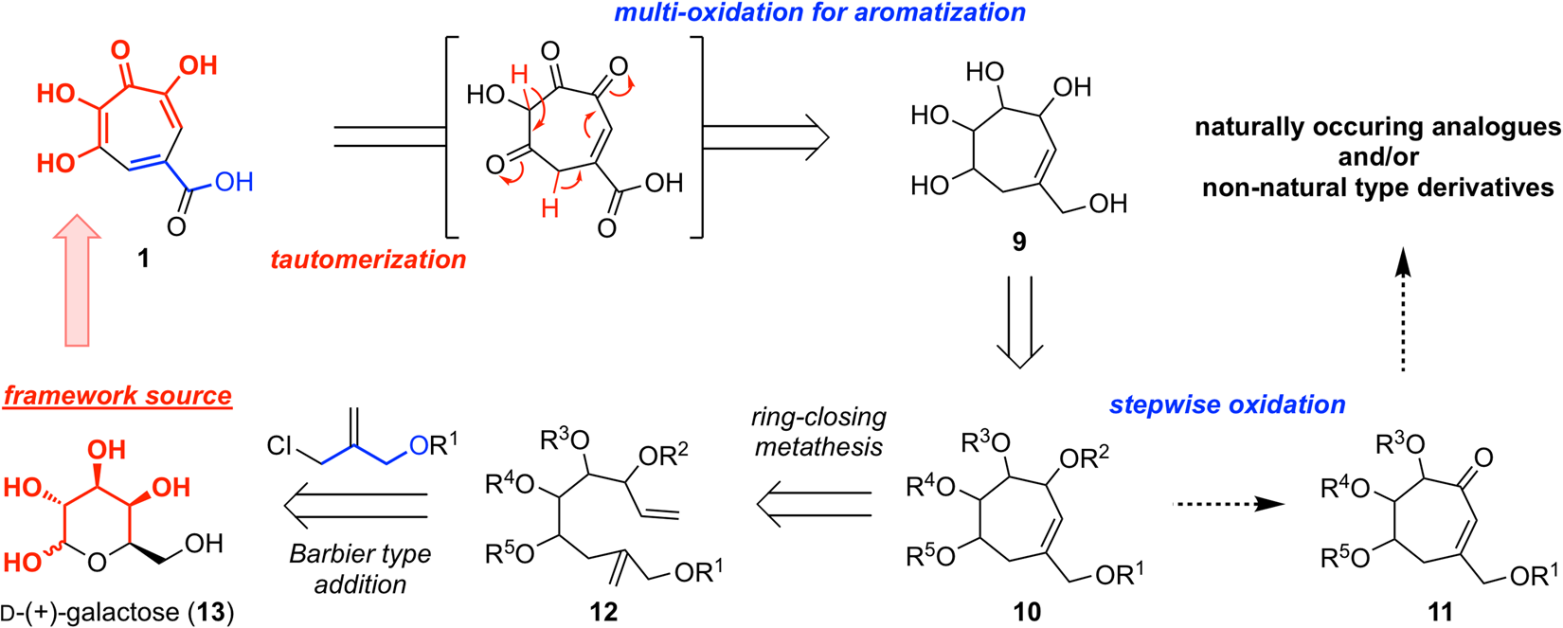
Synthetic strategy for divergent assembly of puberulic acids.

In contrast with synthetic strategy for puberulic acid (**1**)^16, 17^, we proposed an original divergent synthetic route to efficiently produce various analogues and novel derivatives to help clarify the SAR (Fig. 2). A critical point in the synthesis of this class of compounds is fabrication of the 7-membered aromatic ring^18^. We envisaged that the unique highly-oxygenated tropolone framework of **1** could be constructed by multi-oxidation of the 7-membered aliphatic polyalcohol **9** via simultaneous tautomerization and aromatization. Stepwise oxidation of the 7-membered compound **10**, followed by subsequent aromatization, might also allow access to naturally occurring analogues and/or non-natural type derivatives through functionalizations facilitated by the enone **11**. The cyclic compound **10** could be synthesized by functionally-tolerated ring-closing metathesis of the diene **12**, which could be obtained from D-(+)-galactose (**13**) containing the C-C and C-O bonds in the backbone of the target compound by a mild Barbier type addition of allyl chloride^19^. With an efficient supply of compounds established, we overlapped the characteristic highly-oxygenated structure of **1**, with a sugar as one of the cheapest and unlimited natural sources, and chose **13** as a starting material. Although sugars are generally used in syntheses of complex molecules in the chiral pool method from 3-dimensional information^20, 21^, in this synthesis we focused on structural information to utilize the sugar as a “framework source”. This should lead to the target compound using minimal bond-forming reactions^22, 23^.

### Synthesis and oxidation of diol 17 and tetraol 18

**Figure 3:**
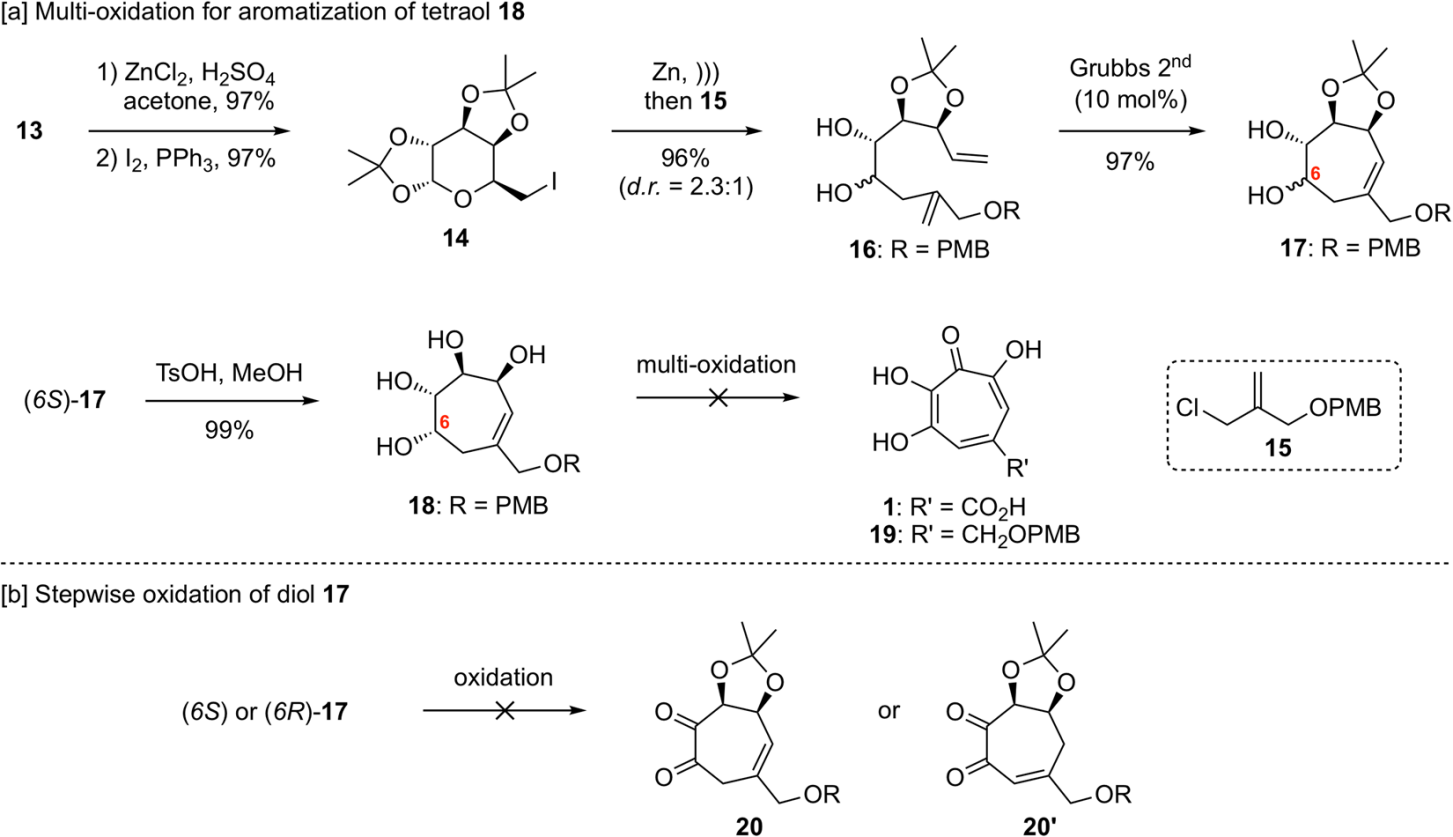
Oxidation of hydroxyl groups on the 7-membered ring.

Manipulation of protection of the hydroxyl groups on the 6-membered ring of **13** and Appel reaction of the resulting primary alcohol, afforded the iodide **14** ^24^. The diene **16** was made possible by the Barbier type addition of the allylchloride **15** ^25^ with **14**, in the presence of zinc dust, providing the desired compound in good yield. Subsequently, ring-closing metathesis of **16**, using Grubbs 2^nd^ catalyst (10 mol%) under high dilution condition (0.01 M), afforded the cyclic compound **17** in excellent yield. Using the major diastereomer (*6S*)-**17**, deprotection of the acetonide group under an acidic condition produced the desired tetraol **18** (Fig. 3a). With respect to investigations of multi-oxidation, we assumed that the PMB group of **18** might be removed under acidic oxidation conditions (e.g. Jones oxidation) or undergo oxidative cleavage (e.g. DDQ or CAN), directly providing **1** or the corresponding aldehyde. However, efforts were unsuccessful (see Supplementary Table S2 in detail) owing to difficulties of monitoring and handling. Consequently, general DMSO-mediated oxidation conditions (e.g. Swern or Parikh-Doering oxidation) or hyper valent iodine reagents (e.g. IBX, DMP or IBS^26, 27^) were attempted with expectations that the PMB ether moiety would remain. In some of these reaction conditions, the loss of two molecules of the hydroxyl group from the desired compound **19** was observed, as determined by mass spectrometry analysis, suggesting that aromatization by β-elimination of oxygen functions occurred when the allylic hydroxyl group on the 7-membered ring was oxidized. Although we considered that stepwise oxidation of diol **17** might be beneficial to obtain clues about multi-oxidation, this transformation was unsuccessful under any conditions (see Supplementary Table S3), probably due to problematic isomerization when the homoallyl alcohol was oxidized and β-elimination of oxygenated functions (Fig. 3b).

### Total synthesis of puberulic acid and viticolin A

**Figure 4:**
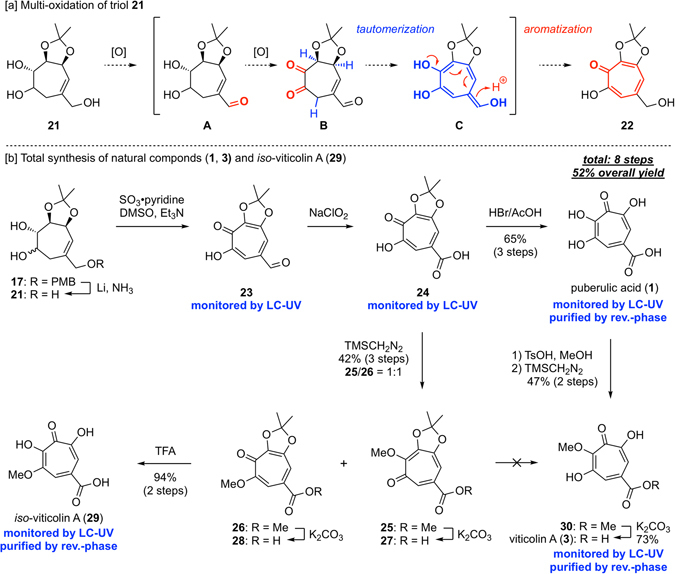
Aromatization strategy and total synthesis of natural products.

As a result of our initial investigations, we turned our attention to the exo-cyclic oxygen atom and envisaged that in multi-oxidation of the triol **21**, the allylic primary alcohol would be oxidized first, providing the α,β-unsaturated aldehyde **A** as a stable intermediate, thus avoiding problematic isomerization and β-elimination. The diol moiety of the resultant **A** might then be further oxidized to generate the triketone **B**, which could undergo tautomerization to produce the enol **C**. Aromatization of **C** would immediately occur by protonation, affording the tropolone **22** (Fig. 4a).

Although removal of the PMB group of the diol **17** was troublesome due to the resulting triol **21** being water-soluble and the condition using DDQ sometimes causing decomposition, it was found that Birch reduction with an unusual work-up procedure reproducibly afforded the desired compound in good yield. With the desired **21** in hand, several oxidation conditions were tried (see Supplementary Table S4). Consequently, we found that treatment of **21** under the Parikh-Doering condition caused multi-tandem oxidation, including additional reaction of **22** to the corresponding aldehyde **23**, which allowed us a fewer step synthesis of the target compound. Finally, Pinnick oxidation of **23** and subsequent removal of the acetonide group afforded puberulic acid (**1**) in 8 steps, with 52% overall yield (Fig. 4b), enabling a practical and scalable total synthesis of **1** ^15^.

We synthesized compounds **25** and **26** during the total synthesis of **1** to detect the generation of the carboxylic acid **24** which was unable to be purified by silica gel due to chelate ability with metals^28^. Although all compounds constructing the tropolone framework (but not the tropone framework) could not be monitored and purified by silica gel, it was found that these compounds could be monitored using LC-UV analysis and purified by reverse-phase chromatography, enabling reliable supply of this class of compounds. We envisioned that the methylated compounds would be valuable to clarify more detailed SAR induced by the information from natural products **3** and **4**, and exploited them for further derivatization. Hydrolysis of the methyl ester in both **25** and **26** afforded two methoxy carboxylic acids **27** and **28**, respectively. Interestingly, removal of the acetonide group of **28** proceeded with 80% TFA at 120 °C in a sealed tube to provide *iso*-viticolin A (**29**) in 94% yield over 2 steps. However, we failed to obtain the natural product, viticolin A (**3**), because the methyl group of its regioisomer **27** was removed faster than the acetonide group under any acidic conditions. We therefore decided to produce natural products **3** and **4** from puberulic acid (**1**) as the starting point. Treatment of **1** with MeOH in the presence of TsOH, followed by careful methylation with TMSCHN_2_, afforded the mono-methylated methylester **30** in 47% yield as a single regioisomer, along with the recovered starting material. We supposed that the inner hydroxyl group of **1** is partly anionic and thus more nucleophilic than the two outer hydroxyl groups due to delocalization of the tropolone framework. It, hence, selectively underwent the methylation allowing access to the natural product. Finally, total synthesis of **3** was accomplished by hydrolysis of **30** in 73% yield. On the other hand, direct methylation of **1** using two equivalents of TMSCHN_2_ gave a complex mixture of the starting material, mono-, di-, and possibly tri-methylated products, as well as stepwise methylation of **30**. In these reactions, viticolin B was detected by LC-UV analysis and^1^H NMR but no selectivity was observed, and thus the given mixture was unable to be separated even by HPLC.

With respect to bioactivity, we first intended to explore the SAR of hydroxyl groups on the tropolone ring which might possibly intensify previously reported bioactivity of the natural compounds (**1**, **2**) and simple troponoids (**5**–**8**)^10, 11^. Since the SAR of **1**, **2**, **6** and **7** indicated that more than three contiguous oxygen atoms on the tropolone ring seem to increase antimalarial activity, we planned to synthesize 7-hydroxytropolone-type derivatives and envisioned that stepwise oxidation of a total synthetic intermediate might allow access to different substituted-type derivatives from **2**.

### Synthesis of 7-hydroxytropolones

**Figure 5:**
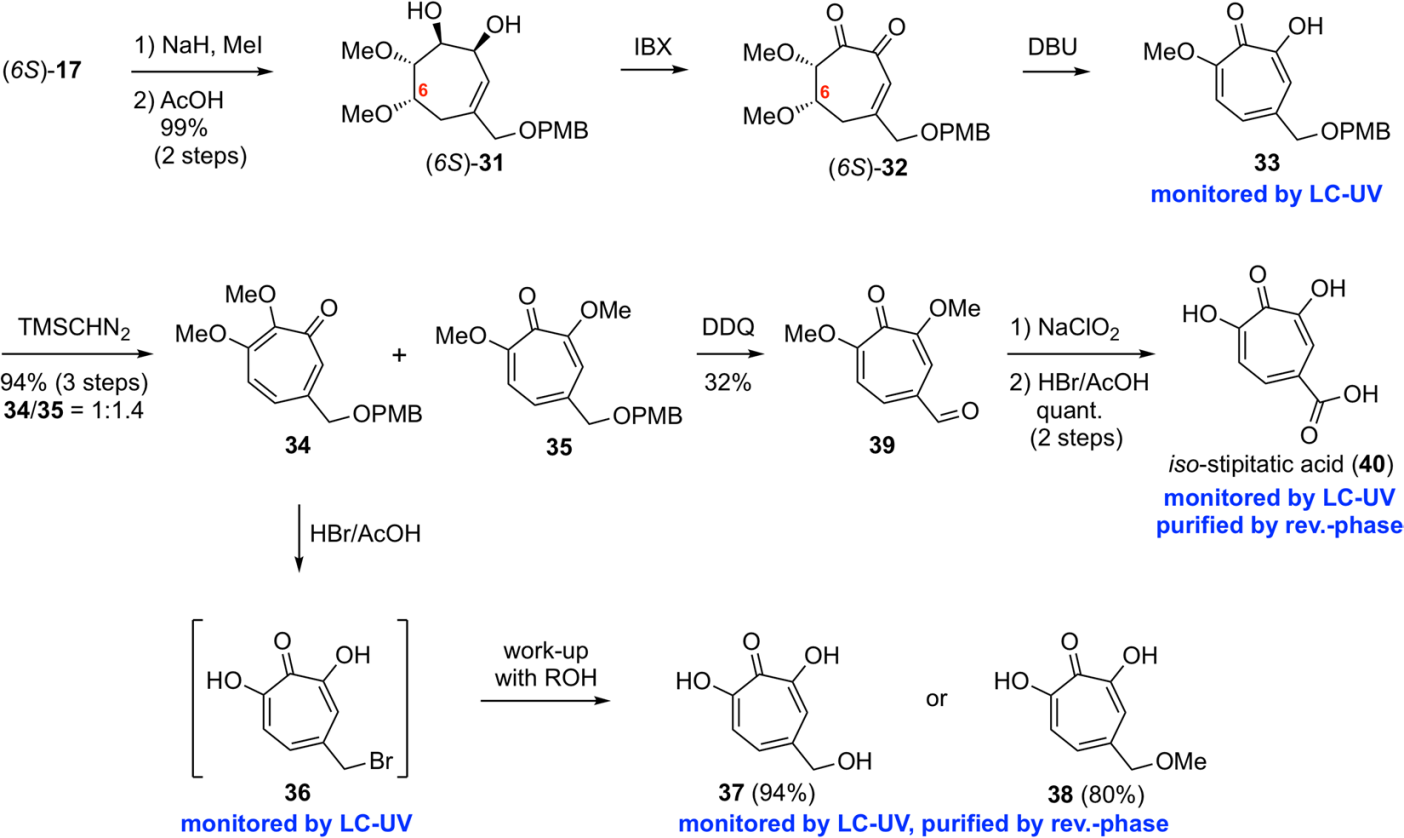
Synthesis of 7-hydroxytropolones.

With the observation of stepwise oxidation of the total synthetic intermediate, diol **17**, we envisaged that 7-hydroxytropolones could be synthesized by aromatization through β-elimination of an alkoxide (Fig. 5). As aforementioned, because diol oxidation was unsuccessful under any conditions, the protective group of (*6S*)-**17** was converted from the acetonide group to the methyl groups in a 2-step manipulation, methylation of the hydroxyl groups and removal of the acetonide group under an acidic condition, providing (*6S*)-**31** in 99% yield. Finally, oxidation of the vicinal diol with IBX proceeded smoothly to afford the diketone (*6S*)-**32**, which was then treated with DBU occurring aromatization by β-elimination to provide the 7-methoxytropolone **33**. For purification, the hydroxyl group of **33** was protected with a methyl group by TMSCHN_2_ to give tropones **34** and **35** as a 1:1.4 separable mixture of the regioisomers in 94% yield over 3 steps. The structure of these compounds was determined by NOE analysis. Using the minor isomer **34**, simultaneous removal of the methyl groups and bromination of the PMB-ether moiety was found to occur following treatment with 33% HBr/AcOH in the presence of H_2_O, providing the bromide **36**, which was then converted to the corresponding alcohol **37** in 94% yield, or methyl ether **38** in 80% yield, by working-up with H_2_O or MeOH, respectively. Conversely, when the major isomer **35** was treated with DDQ, concurrent deprotection of the PMB group and oxidation occurred to provide the aldehyde **39** in a single step. Subsequent oxidation to the carboxylic acid under the Pinnick condition, followed by removal of the methyl groups with HBr/AcOH, afforded the desired *iso*-stipitatic acid (**40**) in a quantitative yield over 2 steps. In this way, we successfully prepared three non-natural 7-hydroxytropolones.

### Bioactivity of tropolone derivatives

Synthesized derivatives, including intermediate compounds, were evaluated for *in vitro* antimalarial activity against the *Plasmodium falciparum* K1 (chloroquine-resistant) parasite strain and for cytotoxicity against a human lung fibroblast cell line MRC-5 (Table 1). We found that synthetic puberulic acid (**1**) showed a similar IC_50_ value (0.044 µM) to that of natural **1**. All intermediates possessing the acetonide group, such as **23**–**28**, did not show any antiparasitic activity, whereas 7-hydroxytropolones, such as **37**, **38**, and **40**, were active (IC_50_ = 4.33, 2.09 and 2.12 µM, respectively), indicating that free hydroxyl groups on the 7-membered ring seem to bestow potency. Although introduction of the methylene hydroxyl group to the C-4 position of **7** did not affect the activity, IC_50_ values of compounds **38** and **40** were approximately 2-fold better than those of **7** and **37**. However, compounds **37** and **38** possessing the methyleneoxy group showed strong cytotoxicity (IC_50_ values of 3.39 and 1.32 nM, respectively), suggesting that substituents at the C-4 position might critically affect cytotoxicity. Remarkably, comparing **40** with stipitatic acid (**2**), the substitution pattern on the tropolone ring seems to be very important. In addition, from the SAR of 7-hydroxytropolones **37**, **38** and **40**, it was found that the carboxyl group is not indispensable for activity. *Iso*-viticolin A (**29**) showed approximately 100-fold stronger activity than viticolin A (**3**). The SAR among natural products **3**, **4** and **29** suggests that three contiguous oxygen atoms with no substitutions increase antiparasitic activity.

**Table 1:** *In vitro* antimalarial activity against *Plasmodium falciparum* K1 strain and cytotoxicity against MRC-5 cells of total synthetic intermediates, 7-hydroxytropolones and methylated derivatives.

Compound	IC_50_ (μM)	
	Antimalarial activity (K1 strain)	Cytotoxicity (MRC-5)
**1** (synthetic)	0.044 ± 0.0029	288.70
**23**	>56	ND*
**24**	>50	ND*
**25**	>47	ND*
**26**	>47	ND*
**27**	>50	ND*
**28**	>50	ND*
**29**	0.34 ± 0.022	55.85
**37**	4.33 ± 0.030	0.0034
**38**	2.09 ± 0.027	0.0013
**40**	2.12 ± 0.11	102.40

*ND: not determined.

Since the SAR information suggested that tetra-substituted tropones with at least three consecutive free-hydroxyl groups and a carbonyl group might have more potent antiparasitic activity, in addition to the convertible carboxylic acid moiety, we next planned to synthesize 6,7-dihydroxytropolones via conversion of the carboxyl group.

### Synthesis of 6,7-dihydroxytropolones

**Figure 6:**
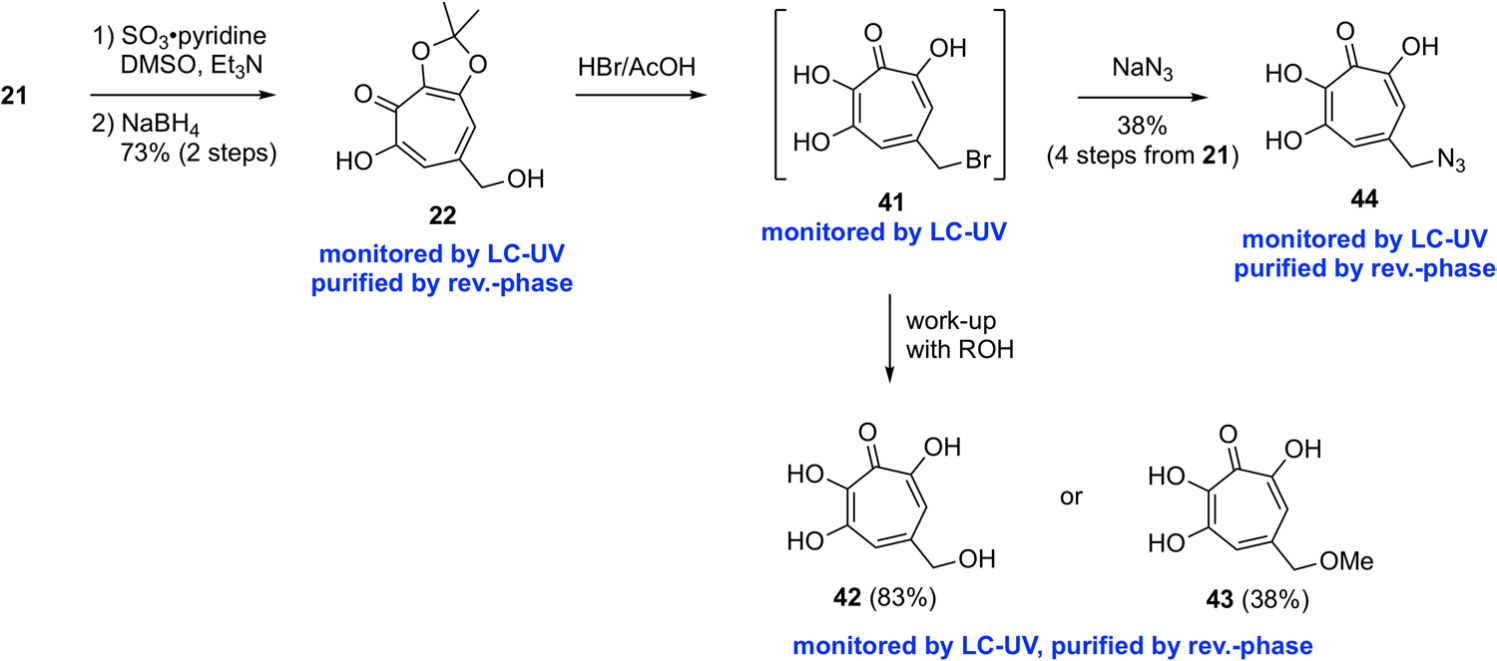
Synthesis of 6,7-dihydroxytropolones.

Varying the carboxyl group of **1**, we envisioned that the same method to synthesize derivatives **30** and **31** could be applicable for transformation of the carboxylic acid moiety (Fig. 6). The total synthetic intermediate, triol **21**, was subjected to multi-tandem oxidation and a subsequent reduction condition using NaBH_4_ to afford the alcohol **22** in 73% yield over 2 steps. Then, treatment of **22** with 33% HBr/AcOH in the presence of H_2_O caused simultaneous removal of the acetonide group and bromination to provide the bromide **41**. This intermediate was converted to the alcohol **42**, in 83% yield, or methyl ether **43**, in 38% yield, by working-up with H_2_O or MeOH, respectively. Furthermore, **41** was found to undergo azidation by treatment with NaN_3_ to afford the azide **44** in 38% yield over 4 steps from triol **21**. This compound would be useful for further derivatization using Click chemistry^29^.

### Bioactivity of 6,7-dihydroxytropolones

*In vitro* antimalarial activity and cytotoxicity of synthesized 6,7-dihydroxytropolones **42**–**44** were evaluated, as shown in Table 2. The alcohol **42** and the methyl ether **43** showed the same order of activity as natural **1**. Although the azide **44** exhibited weaker activity compared with **42** and **43**, all these derivatives were approximately 10-fold more potent than the 7-hydroxytropolones, suggesting that contiguous four oxygen atoms on the tropolone ring are important for the antiparasitic property. In addition, all these compounds showed cytotoxicity. Our result indicated that this class of compounds, with potent antimalarial activity, tends to show strong cytotoxicity in human cells as well.

**Table 2:** *In vitro* antimalarial activity and cytotoxicity of 6,7-dihydroxytropolones.

Compound	IC_50_ (μM)	
	Antimalarial activity (K1 strain)	Cytotoxicity (MRC-5)
**42**	0.21 ± 0.0033	0.024
**43**	0.22 ± 0.0016	0.091
**44**	0.46 ± 0.053	0.0035

These results will be useful in helping to explore more promising drug leads for malaria, and more detailed bioactivity evaluation of synthesized derivatives in a mouse model are now ongoing.

## Conclusions

Inspired by the SAR information of natural products isolated by our group and the apparent antiparasitic activity of simple troponoids, we divergently synthesized troponoids using intermediates obtained during the established method for synthesizing puberulic acid. Synthesis of the 7-hydroxytropolones was accomplished by stepwise oxidation-aromatization sequence via β-elimination, which allowed access to non-natural derivatives. Conversion of the carboxylic acid moiety in the natural products was accomplished via brominated key intermediates generated by treatment with HBr/AcOH. This method was applicable to convert the bromide to nucleophiles, which allowed divergent production of derivatives. *In vitro* antimalarial activity and cytotoxicity of synthesized derivatives were evaluated and several derivatives were found with the same order of antiparasitic activity as the potent natural product, puberulic acid. The SAR information we obtained will be invaluable for exploration of more promising candidates for antimalarial drugs and further derivatization of this class of compounds, and further analysis of the bioactivity of these chemicals, is currently in progress.

## Methods

### General information

Unless otherwise noted, reagents and solvents were purchased at the highest commercial quality and used without further purification. LC-UV analysis was carried out with Agilent 1100 system (Agilent Technology, Inc.) under the following condition; column, Symmetry C18 (Waters Co., Ltd., 2.1 φ × 150 mm); UV detection, 210 nm; flow rate, 0.2 mL/min; mobile phase, MeCN-H_2_O with 0.05% H_3_PO_4_, (5–100% linear gradient over 20 min). ODS column chromatography was carried out with Sep-Pak^®^ Plus C18 Short Cartridge (Waters Co., Ltd.) or CHROMATOREX^®^ (Fuji Silysia Chemical, Ltd.). ^1^H NMR spectra were recorded on JEOL JNM-ECA-500 (500 MHz) and ^13^C NMR spectra were recorded on JEOL JNM-ECA-500 (125 MHz). Chemical shifts are expressed in ppm downfield from the internal solvent peaks for CD_3_OD (^1^H; δ = 3.31 ppm,^13^C; δ = 49.0 ppm) and *J* values are given in Hertz. The following abbreviations were used to explain the multiplicities: s = singlet, d = doublet, and br = broad. High- and Low-resolution mass spectra were measured on JEOL JMS-AX505 HA, JEOL JMS-700 MStation and JEOL JMS-T100LP.

### Viticolin A (3)

To a solution of **30** (7.0 mg, 30.97 µmol) in THF (1.55 mL) and H_2_O (1.55 mL) was added K_2_CO_3_ (21.4 mg, 0.15 mmol) at room temperature. After being stirred at 50 °C for 1 d, H_2_O (20 mL) was added to the reaction mixture. The resulting mixture was extracted with CHCl_3_ (20 mL × 2). The aqueous layer was acidified by 1 M HCl, and extracted with CHCl_3_:*i*-PrOH = 10:1 (40 mL × 4). The combined organic layer was dried over sodium sulfate, and concentrated under reduced pressure. The residue was purified by Sep-pak^®^ Plus C18 Short Cartridge to afford **3** (4.8 mg, 73%) as a yellow solid.

^1^H NMR (500 MHz, CD_3_OD) δ7.82 (d, *J* = 1.2 Hz, 1 H), 7.72 (d, *J* = 1.2 Hz, 1 H), 3.95 (s, 3 H); ^13^C NMR (125 MHz, CD_3_OD) δ172.0, 168.7, 163.3, 160.6, 150.0, 134.4, 123.4, 112.0, 59.6; HRMS-ESI (*m/z*) [M–H]^−^ calcd for C_9_H_7_O_6_ 211.0243, found 211.0234; mp 227 °C.

### *Iso*-viticolin A (29)

To a solution of **26** (10.7 mg, 40.19 µmol) in MeOH (0.40 mL), H_2_O (0.40 mL), and THF (0.40 mL) was added K_2_CO_3_ (27.8 mg, 0.20 mmol) at room temperature. After being stirred at room temperature for 5 h, H_2_O (20 mL) was added to the reaction mixture. The resulting mixture was extracted with CHCl_3_ (20 mL) The aqueous layer was acidified by 1 M HCl, and extracted with CHCl_3_:*i*-PrOH = 10:1 (40 mL × 3). The combined organic layer was dried over sodium sulfate, and concentrated under reduced pressure to yield the crude product as a yellow solid. This crude product was used in the next reaction without further purification.

To the crude product in a sealed tube was added 80% TFA aq. (0.40 mL) at room temperature. After being stirred at 120 °C for 6 h, the reaction mixture was concentrated under reduced pressure. The residue was purified by Sep-pak^®^ Plus C18 Short Cartridge to afford **29** (8.0 mg, 94% over 2 steps) as a yellow solid.

^1^H NMR (500 MHz, CD_3_OD) δ8.01 (s, 1H), 7.96 (s, 1H), 4.03 (s, 3H); ^13^C NMR (125 MHz, CD_3_OD) δ169.6, 162.7, 160.7, 157.4, 156.7, 128.8, 121.6, 116.4, 57.3; HRMS-ESI (*m/z*) [M–H]^−^ calcd for C_9_H_7_O_6_ 211.0243, found 211.0240; mp 183 °C (decomp.).

### 4-Hydroxymethyl-7-hydroxytropolone (37)

To **34** (6.0 mg, 18.97 µmol) in a sealed tube was added a 4:1 mixture of 33% HBr/AcOH and H_2_O (0.19 mL) at room temperature. After being stirred at 120 °C for 2 h, the reaction mixture was concentrated under reduced pressure. The residue was purified by Sep-pak^®^ Plus C18 Short Cartridge using acetone/H_2_O to afford **37** (3.0 mg, 94%) as a yellow solid.

^1^H NMR (500 MHz, CD_3_OD) δ7.73 (s, 1H), 7.60 (d, *J* = 10.9 Hz, 1H), 7.42 (d, *J* = 10.9 Hz, 1H), 4.64 (s, 2H); ^13^C NMR (125 MHz, CD_3_OD) δ168.9, 161.5, 160.8, 144.5, 127.9, 122.0, 121.2, 67.2; HRMS-EI (*m/z*) [M^+^] calcd for C_8_H_8_O_4_ 168.0423, found 168.0426; mp 144 °C (decomp.).

### 4-Methoxymethyl-7-hydroxytropolone (38)

To **34** (5.0 mg, 15.81 µmol) in a sealed tube was added a 4:1 mixture of 33% HBr/AcOH and H_2_O (0.16 mL) at room temperature. After being stirred at 120 °C for 2 h, the reaction mixture was concentrated under reduced pressure. The residue was purified by Sep-pak^®^ Plus C18 Short Cartridge using MeOH/H_2_O to afford **38** (2.3 mg, 80%) as a yellow oil.

^1^H NMR (500 MHz, CD_3_OD) δ7.48 (br-s, 1H), 7.41 (d, *J* = 10.9 Hz, 1H), 7.18 (br-d, *J* = 10.9 Hz, 1H), 4.42 (s, 2H), 3.40 (s, 3H); ^13^C NMR (125 MHz, CD_3_OD) δ169.5, 161.4, 161.2, 141.1, 129.0, 121.73, 121.68, 77.7, 58.5; HRMS-EI (*m/z*) [M^+^] calcd for C_9_H_10_O_4_ 182.0579, found 182.0572.

### *Iso*-stipitatic acid (40)

To a solution of **39** (4.0 mg, 20.60 µmol) and 2-methyl-2-butene (43.77 µL, 0.41 mmol) in THF (0.21 mL) and *t*-BuOH (0.21 mL) was added the mixture of NaClO_2_ (2.8 mg, 30.90 µmol) and NaH_2_PO_4_ (9.6 mg, 61.80 µmol) in H_2_O (0.21 mL) dropwise by a Pasteur pipette at room temperature. After being stirred at room temperature for 30 min, the reaction mixture was directly filtrated by silica gel, eluted with CHCl_3_/MeOH = 10:1, and concentrated under reduced pressure to afford the crude product as a yellow solid. This crude product was used in the next reaction without further purification.

To the crude product in a sealed tube was added a 4:1 mixture of 33% HBr/AcOH and H_2_O (0.42 mL) at room temperature. After being stirred at 120 °C for 2 h, the reaction mixture was concentrated under reduced pressure. The residue was purified by Sep-pak^®^ Plus C18 Short Cartridge to afford **40** (3.8 mg, quant. over 2 steps) as a yellow solid.

^1^H NMR (500 MHz, CD_3_OD) δ8.15 (s, 1H), 8.06 (d, *J* = 10.9 Hz, 1H), 7.45 (d, *J* = 10.9 Hz, 1H); ^13^C NMR (125 MHz, CD_3_OD) δ171.7, 169.2, 164.3, 159.9, 132.3, 130.3, 120.5, 119.5; HRMS-ESI (*m/z*) [M–H]^−^ calcd for C_8_H_5_O_5_ 181.0137, found 181.0128; mp 170 °C (decomp.).

### 4-Hydroxymethyl-6,7-dihydroxytropolone (42)

With the same procedure for the synthesis of **37**, **42** (9.8 mg, 83%) was obtained from **22** (14.4 mg, 64.23 µmol) as a yellow solid.

^1^H NMR (500 MHz, CD_3_OD) δ 7.15 (s, 2H), 4.52 (s, 2H); ^13^C NMR (125 MHz, CD_3_OD) δ 158.4, 157.2, 143.8, 117.2, 67.3; HRMS-EI (*m/z*) [M^+^] calcd for C_8_H_8_O_5_ 184.0372, found 184.0388; mp 162 °C.

### 4-Methoxymethyl-6,7-dihydroxytropolone (43)

With the same procedure for the synthesis of **38**, **43** (1.7 mg, 38%) was obtained from **22** (5.1 mg, 22.75 µmol) as a yellow solid.

^1^H NMR (500 MHz, CD_3_OD) δ7.10 (s, 2H), 4.38 (s, 2H), 3.40 (s, 3H); ^13^C NMR (125 MHz, CD_3_OD) δ158.4, 157.2, 140.1, 117.8, 77.6, 58.3; HRMS-ESI (*m/z*) [M–H]^−^ calcd for C_9_H_9_O_5_ 197.0450, found 197.0450; mp 120 °C.

### 4-Azidomethyl-6,7-dihydroxytropolone (44)

With the same procedure for the synthesis of **42** without purification, **41** was obtained as a intermediate from (*6S*)-**17** (21.4 mg, 92.93 µmol) as a dark brown amorphous. This crude product was used in the next reaction without further purification.

To a solution of the crude **41** in DMF (0.93 mL) was added NaN_3_ (18.1 mg, 0.28 mmol) at room temperature. After being stirred at room temperature for 2.5 h, the reaction mixture was quenched with H_2_O (10 mL). The organic layer was separated, and the aqueous layer was extracted with EtOAc (10 mL × 3). The combined organic layer was dried over sodium sulfate, and concentrated under reduced pressure. The residue was purified by Sep-pak^®^ Plus C18 Short Cartridge to afford **44** (7.4 mg, 38% over 4 steps) as a yellow solid.

^1^H NMR (500 MHz, CD_3_OD) δ7.09 (s, 2H), 4.35 (s, 2H); ^13^C NMR (125 MHz, CD_3_OD) δ158.0, 157.6, 137.3, 118.8, 58.8; HRMS-ESI (*m/z*) [M–H]^−^ calcd for C_8_H_6_O_4_N_3_ 208.0358, found 208.0358; mp 159 °C.

*Experimental details of other new compounds and additional information are given in the Supplementary Information.

### *In vitro* cultivation of *Plasmodium falciparum* and the antimalarial assay

*In vitro* cultivation and antimalarial activity against the *Plasmodium falciparum* K1 (chloroquine-resistant) parasite strain were measured using the method described previously^30^. Briefly, the *P. falciparum* K1 strain was cultured in human erythrocytes in RPMI medium supplemented with 10% human plasma at 37 °C, under 93% N_2_, 4% CO_2_, and 3% O_2_. Asynchronous parasites (2% hematocrit and 0.5 or 1% parasitaemia) were seeded in a 96-well microtiter plate and serially diluted test compounds were added. Positive controls, such as chloroquine and artemisinin, were added in a similar fashion. After 72-hours incubation, parasite lactate dehydrogenase (p-LDH) was assayed using a slight modification of the procedure reported by Makler *et al*.^31^ and Vivas *et al*.^32^. The 50% inhibitory concentration (IC_50_) value was calculated from a dose response curve. This study was approved by “Kitasato Institute Hospital Research Ethics Committee (No12102)” using human erythrocytes donated by volunteers.

### Cytotoxic assay against MRC-5 cells

Measurement of cytotoxicity against human fetal lung fibroblast MRC-5 cells was carried out as described previously^33^.

## Electronic supplementary material

Supplementary Information
