# Supplementary material for: Antimalarial troponoids, puberulic acid and viticolins; divergent synthesis and structure-activity relationship studies
Source: Sci Rep. 2017 Aug 3;7:7259. doi: 10.1038/s41598-017-07718-3 (PMC5543150; doi:10.1038/s41598-017-07718-3)
Supplement: Supplementary file 1 — Supplementary Information [file 41598_2017_7718_MOESM1_ESM.pdf]

Antimalarial troponoids, puberulic acid and viticolins; divergent synthesis and structure-activity relationship studies

Supplementary Information

Goh Sennari, Ryo Saito, Tomoyasu Hirose, Masato Iwatsuki,
Aki Ishiyama, Rei Hokari, Kazuhiko Otoguro,
Satoshi Ōmura and Toshiaki Sunazuka

Graduate School of Infection Control Sciences, Kitasato University
Kitasato Institute for Life Sciences, Kitasato University
5-9-1 Shirokane, Minato-ku, Tokyo 108-8641, Japan.

1. General Methods and Material -S2-
2. Experimental Procedures and Compounds Characterization -S3-
    2-1. Synthesis of viticolins -S3-
    2-2. Synthesis of 7-hydroxytropolones -S6-
    2-3. Synthesis of 6,7-dihydroxytropolones -S11-
    2-4. Data comparison of synthetic viticolin A with the natural product -S14-
3. Biological evaluation of synthesized derivatives -S17-
4. Purity assessment of evaluated derivatives -S19-
5. Reaction screenings -S20-
    Table S2. Multi-oxidation of tetraol (*6S*)-**18** -S20-
    Table S3. Stepwise oxidation of diol **17** -S21-
    Table S4. Multi-oxidation of triol **21** -S22-
6. Spectra Charts -S23-

1. General Methods and Materials

Unless otherwise noted, reagents and solvents were purchased at the highest commercial quality and used without further purification. Dry DMF, THF, CH_2Cl_2, MeOH, PhH, *t*-BuOH, and DMSO were purchased from Kanto Chemical Co., Inc. Pre-coated silica gel plates with a fluorescent indicator (Merck 60 F254) were used for analytical (0.25 mm) and preparative (0.25 or 0.50 mm) thin layer chromatography. LC-UV analysis was carried out with Agilent 1100 system (Agilent Technology, Inc.) under the following condition; column, Symmetry C18 (Waters Co., Ltd., 2.1 φ × 150 mm); UV detection, 210 nm; flow rate, 0.2 mL/min; mobile phase, MeCN-H_2O with 0.05% H_3PO_4, (5-100% linear gradient over 20 min). Flash chromatography was carried out with Kanto Chemical silica gel (Kanto Chemical, silica gel 60N, spherical neutral, 0.040–0.050 mm, Cat.-No. 37563–84) or Merck silica gel 230-400 mesh ASTM (60N, 0.040-0.063 mm, Cat.-No. 109385). ODS column chromatography was carried out with Sep-Pak® Plus C18 Short Cartridge (Waters Co. Ltd.) or CHROMATOREX® (Fuji Silysia Chemical Ltd.). ^{1}H NMR spectra were recorded on JEOL JNM-ECA-500 (500 MHz) and ^{13}C NMR spectra were recorded on JEOL JNM-ECA-500 (125 MHz). Chemical shifts are expressed in ppm downfield from the internal solvent peaks for $CDCl_3$ (^{1}H; δ = 7.26 ppm, ^{13}C; δ = 77.0 ppm), CD_3OD (^{1}H; δ = 3.31 ppm, ^{13}C; δ = 49.0 ppm), and $(CD_3)_2CO$ (^{1}H; δ = 2.05 ppm, ^{13}C; δ = 29.8, 206.3 ppm) and *J* values are given in Hertz. The following abbreviations were used to explain the multiplicities: s = singlet, d = doublet, dd = double doublet, ddd = double double doublet, m = multiplet, br = broad. lnfrared spectra for all compounds were measured on a HORIBA FT-210 spectrometer. High- and Low-resolution mass spectra were measured on a JEOL JMS-AX505 HA, JEOL JMS-700 MStation and JEOL JMS-T100LP. Optical rotations were measured by using JASCO P-1010 polarimeter.

2. Experimental Procedures and Compounds Characterization

2-1. Synthesis of viticolins

Iso-viticolin A (**29**)

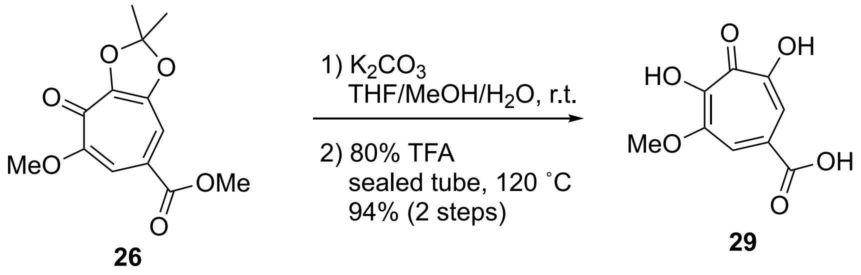

To a solution of **26** (10.7 mg, 40.19 μmol) in MeOH (0.40 mL), H_2O (0.40 mL), and THF (0.40 mL) was added K_2CO_3 (27.8 mg, 0.20 mmol) at room temperature. After being stirred at room temperature for 5 h, H_2O (20 mL) was added to the reaction mixture. The resulting mixture was extracted with $CHCl_3$ (20 mL) The aqueous layer was acidified by 1M HCl, and extracted with $CHCl_3$/*i*-PrOH = 10:1 (40 mL × 3). The combined organic layer was dried over sodium sulfate, and concentrated under reduced pressure to yield the crude product as a yellow solid. This crude product was used in the next reaction without further purification.

To the crude product in a sealed tube was added 80% TFA aq. (0.40 mL) at room temperature. After being stirred at 120 ˚C for 6 h, the reaction mixture was concentrated under reduced pressure. The residue was purified by Sep-pak® Plus C18 Short Cartridge to afford **29** (8.0 mg, 94% over 2 steps) as a yellow solid.

^{1}H NMR (500 MHz, CD_3OD) δ 8.01 (s, 1H), 7.96 (s, 1H), 4.03 (s, 3H); ^{13}C NMR (125 MHz, CD_3OD) δ 169.6, 162.7, 160.7, 157.4, 156.7, 128.8, 121.6, 116.4, 57.3; IR (Diamond prism) ν_{max} 3714, 3648, 3621, 3575, 3370, 3270, 3208, 2541, 2437, 2194, 2163, 2098, 2036, 1967, 1897, 1442, 1203, 1076, 968, 698, 582, 497, 455 cm^{-1}; HRMS-ESI (*m/z*) $[M–H]^-$ calcd for $C_9H_7O_6$ 211.0243, found 211.0240; mp 183 ˚C (decomp.)

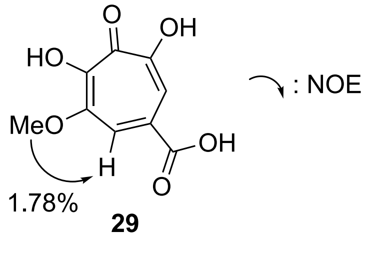

Carboxylic acids **27** and **28**

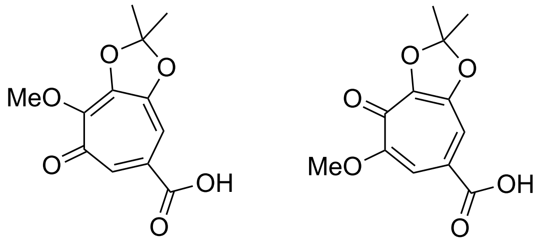

27　　**28**

According to the procedure for preparation of **29**, hydrolysis of methylesters **25** or **26** afforded carboxyli acids **27** as a pale yellow solid, or **28** as a pale yellow solid, respectively.

27: ^{1}H NMR (500 MHz, CD_3OD) δ 7.67 (s, 1H), 7.21 (s, 1H), 3.93 (s, 3H), 1.79 (s, 6H); ^{13}C NMR (125 MHz, CD_3OD) δ 181.4, 156.4, 153.5, 149.7, 134.0, 120.3, 106.9, 60.4, 26.0; IR (Diamond prism) ν_{max} 3494, 2962, 2240, 2194, 2140, 2082, 2044, 1978, 1677, 1604, 1577, 1515, 1430, 1353, 1253, 1222, 1106, 1006, 894, 829, 771, 674, 605; HRMS-ESI (*m/z*) $[M+Na]^+$ calcd for $C_{12}H_{12}O_6Na$ 275.0534, found 275.0528; mp 177 ˚C

28: ^{1}H NMR (500 MHz, CD_3OD) δ 7.88 (s, 1H), 7.82 (s, 1H), 4.03 (s, 3H), 1.79 (s, 6H); ^{13}C NMR (125 MHz, CD_3OD) δ 172.1, 167.0, 164.8, 153.8, 150.6, 141.0, 119.5, 117.0, 113.6, 56.7, 25.8; IR (Diamond prism) ν_{max} 3490, 3301, 2992, 2927, 2838, 2233, 2159, 2105, 1967, 1592, 1554, 1492, 1446, 1396, 1222, 1110, 1076, 998, 952, 817, 674, 582; HRMS-ESI (*m/z*) $[M+Na]^+$ calcd for $C_{12}H_{12}O_6Na$ 275.0532, found 275.0522; mp 113 ˚C (decomp.)

Methylester (**30**)

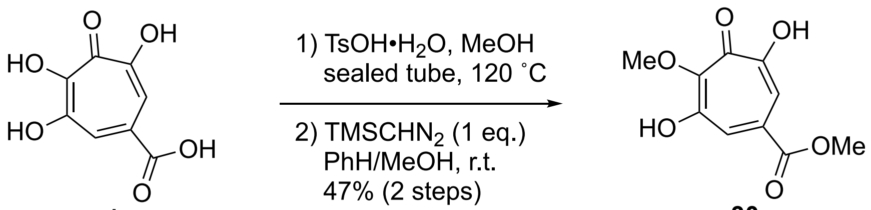

1　　**30**

To **1** (18.2 mg, 91.86 μmol) in a sealed tube was added MeOH (0.92 mL) and TsOH•H_2O (1.8 mg, 9.19 μmol) at room temperature. After being stirred at 120 ˚C for 1 d, the reaction mixture was cooled to room temperature and H_2O (5 mL) was added.

The resulting mixture was extracted with $CHCl_3$ (10 mL × 3), and the combined organic layer was dried over sodium sulfate, and concentrated under reduced pressure to yield crude product as a yellow solid. This crude product was used in the next reaction without further purification.

To a solution of the crude product in PhH (8.37 mL) and MeOH (0.92 mL) was added $TMSCHN_2$ (45.9 μL, 91.86 μmol) at room temperature. After being stirred at room temperature for 1 h, the resulting mixture was concentrated under reduced pressure. The residue was purified by HPLC (20% MeOH/H_2O) to afford **30** (9.7 mg, 47% over 2 steps) as a yellow solid.

^{1}H NMR (500 MHz, CD_3OD) δ 7.78 (s, 1H), 7.67 (s, 1H), 3.95 (s, 3H), 3.94 (s, 3H); ^{13}C NMR (125 MHz, CD_3OD) δ 172.5, 167.8, 163.9, 160.9, 150.7, 133.1, 124.1, 111.8, 60.1, 53.8; IR (Diamond prism) ν_{max} 3367, 3220, 2958, 2507, 2399, 2159, 2051, 2003, 1936, 1727, 1585, 1553, 1484, 1434, 1380, 1303, 1257, 1191, 1149, 1064, 998, 937, 902, 732, 655 cm^{-1}; HRMS-ESI (*m/z*) $[M–H]^-$ calcd for $C_{10}H_9O_6$ 225.0399, found 225.0390; mp 187 ˚C

Viticolin A (**3**)

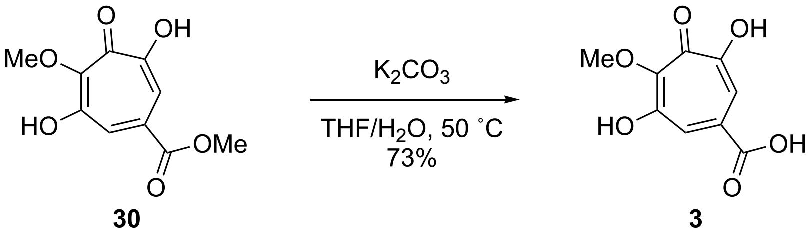

To a solution of **30** (7.0 mg, 30.97 μmol) in THF (1.55 mL) and H_2O (1.55 mL) was added K_2CO_3 (21.4 mg, 0.15 mmol) at room temperature. After being stirred at 50 ˚C for 1 d, H_2O (20 mL) was added to the reaction mixture. The resulting mixture was extracted with $CHCl_3$ (20 mL × 2). The aqueous layer was acidified by 1M HCl, and extracted with $CHCl_3$/*i*-PrOH = 10:1 (40 mL × 4). The combined organic layer was dried over sodium sulfate, and concentrated under reduced pressure. The residue was purified by Sep-pak® Plus C18 Short Cartridge to afford **3** (4.8 mg, 73%) as a yellow solid.

^{1}H NMR (500 MHz, CD_3OD) δ 7.82 (d, *J* = 1.2 Hz, 1H), 7.72 (d, *J* = 1.2 Hz, 1H), 3.95 (s, 3H); ^{13}C NMR (125 MHz, CD_3OD) δ 172.0, 168.7, 163.3, 160.6, 150.0, 134.4, 123.4, 112.0, 59.6; IR (Diamond prism) ν_{max} 3320, 3247, 2962, 2923, 2854, 1970, 1916, 1700,

1573, 1519, 1380, 1292, 1241, 1180, 1022, 941, 906, 798, 728, 690 cm^{-1}; HRMS-ESI (*m/z*) [M–H]$^{-}$ calcd for $C_9H_7O_6$ 211.0243, found 211.0234; mp 227 ˚C

2-2. Synthesis of 7-hydroxytropolones

Diol (*6S*)-**31**

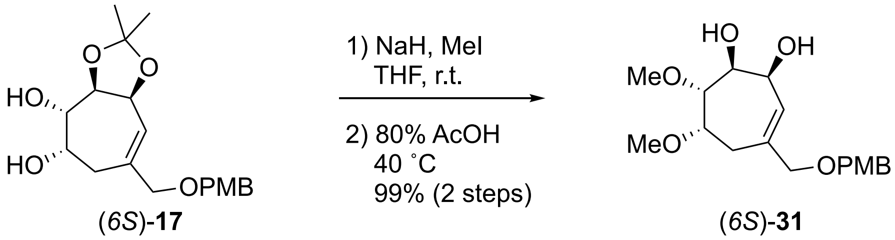

To a solution of (*6S*)-**17** (0.75 g, 2.14 mmol) in THF (21.40 mL) was added NaH (0.43 g, 10.70 mmol) and MeI (0.67 mL, 10.70 mmol) at room temperature. After being stirred at room temperature for 1 h, the reaction mixture was quenched with sat. aq. NH_4Cl (40 mL). The organic layer was separated, and the aqueous layer was extracted with EtOAc (40 mL). The combined organic layer was washed with brine (80 mL), dried over sodium sulfate, and concentrated under reduced pressure to yield the crude product as a brown oil. This crude product was used in the next reaction without further purification.

To the crude product in a flask was added 80% AcOH aq. (4.28 mL) at room temperature. After being stirred at 40 ˚C for 11 h, the reaction mixture was concentrated under reduced pressure. The residue was purified by silica gel flash column chromatography (Hexanes/EtOAc = 1:5) to afford (*6S*)-**31** (0.72 g, 99% over 2 steps) as a colorless oil.

^{1}H NMR (500 MHz, $CDCl_3$) δ 7.25 (d, *J* = 8.6 Hz, 2H), 6.87 (d, *J* = 8.6 Hz, 2H), 5.70 (d, *J* = 5.2 Hz, 1H), 4.65 (d, *J* = 5.2 Hz, 1H), 4.42 (s, 2H), 3.99 (dd, *J* = 7.5, 1.7 Hz, 1H), 3.90 (s, 2H), 3.80 (s, 3H), 3.65 (dd, *J* = 6.9, 1.7 Hz, 1H), 3.57 (ddd, *J* = 9.2, 2.3, 1.7 Hz, 1H), 3.52 (s, 3H), 3.35 (s, 3H), 2.66 (dd, *J* = 14.9, 9.2 Hz, 1H), 2.23 (d, *J* = 14.9 Hz, 1H); ^{13}C NMR (125 MHz, $CDCl_3$) δ 159.4, 137.0, 130.3, 129.5, 128.4, 113.9, 83.0, 76.3, 75.0, 72.8, 71.8, 69.0, 59.0, 57.4, 55.4, 28.1; IR (Diamond prism) ν_{max} 3675, 3556, 2927, 2834, 2210, 2109, 2032, 1986, 1940, 1989, 1612, 1511, 1457, 1357, 1299, 1245, 1172, 1076, 1029, 914, 848, 817, 682 cm^{-1}; HRMS-ESI (*m/z*) [M+Na]$^+$ calcd for $C_{18}H_{26}O_6Na$ 361.1627, found 361.1618; $[\alpha]_D^{24}$ +14.4 (*c* 0.1 MeOH)

Tropones **34** and **35**

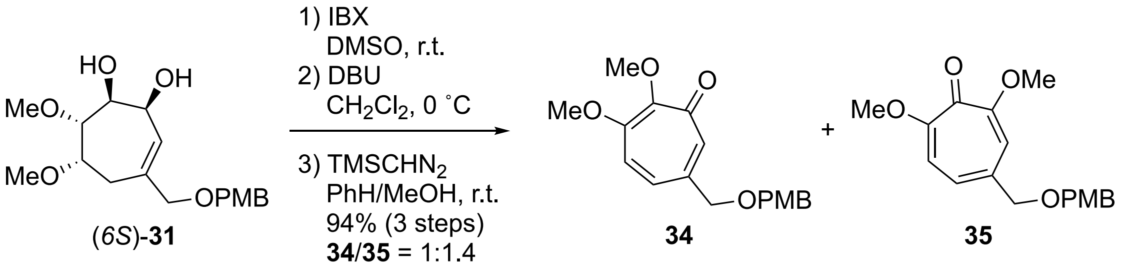

To a solution of (*6S*)-**31** (0.40 g, 1.18 mmol) in DMSO (11.82 mL) was added IBX (0.99 g, 3.55 mmol) at room temperature. After being stirred at room temperature for 10 min, the reaction mixture was quenched with H_2O (20 mL). The resulting suspension was filtrated through a pad of Celite®, washed with EtOAc. The organic layer was separated, and the aqueous layer was extracted with EtOAc (20 mL). The combined organic layer was washed with brine (60 mL), dried over sodium sulfate, and concentrated under reduced pressure to yield the crude product as a yellow oil. This crude product was used in the next reaction without further purification.

To a stirred solution of the crude product in CH_2Cl_2 (11.82 mL) was added DBU (0.53 mL, 3.55 mmol) at 0 ˚C. After being stirred at 0 ˚C for 15 min, the reaction mixture was quenched with 1M HCl (20 mL). The organic layer was separated, and the aqueous layer was extracted with CH_2Cl_2 (20 mL). The combined organic layer was dried over sodium sulfate, and concentrated under reduced pressure to yield the crude product as a yellow oil. This crude product was used in the next reaction without further purification.

To a stirred solution of the crude product in PhH (10.64 mL) and MeOH (1.18 mL) was added $TMSCHN_2$ (0.6M in hexane; 9.85 mL, 5.91 mmol) at room temperature. After being stirred at room temperature for 9 h, the reaction mixture was concentrated under reduced pressure at 20 ˚C. The residue was purified by silica gel flash column chromatography (only EtOAc to $CHCl_3$/MeOH = 20:1) to afford **34** (0.14 g, 39% over 3 steps) as a yellow oil, and **35** (0.21 g, 55% over 3 steps) as a yellow solid.

34: ^{1}H NMR (500 MHz, $(CD_3)_2CO$) δ 7.32 (d, J = 8.6 Hz, 2H), 7.15 (d, J = 12.6 Hz, 1H), 7.09 (br-d, J = 1.2 Hz, 1H), 6.92 (d, J = 8.6 Hz, 2H), 6.90 (dd, J = 12.6, 1.7 Hz, 1H), 4.53 (s, 2H), 4.39 (d, J = 1.2 Hz, 2H), 3.92 (s, 3H), 3.81 (s, 3H), 3.79 (s, 3H); ^{13}C NMR (125 MHz, $(CD_3)_2CO$) δ 180.7, 160.4, 158.5, 156.3, 144.6, 137.6, 131.0, 130.3, 130.2, 128.6, 114.6, 73.5, 72.8, 59.2, 55.5; IR (Diamond prism) ν_{max} 2938, 2838, 2240, 2186, 2078, 2021, 1963, 1739, 1562, 1511, 1461, 1442, 1299, 1245, 1168, 1083, 1029, 991,

948, 817, 767 cm^{-1}; HRMS-ESI (*m/z*) $[M+Na]^+$ calcd for $C_{18}H_{20}O_5Na$ 339.1208, found 339.1207

35: ^1H NMR (500 MHz, $(CD_3)_2CO$) δ 7.32 (d, *J* = 8.6 Hz, 2H), 6.99-6.91 (complex m, 3H), 6.92 (d, *J* = 8.6 Hz, 2H), 4.51 (s, 2H), 4.45 (s, 2H), 3.85 (s, 3H), 3.84 (s, 3H), 3.79 (s, 3H); ^{13}C NMR (125 MHz, $(CD_3)_2CO$) δ 173.6, 162.35, 162.29, 160.3, 136.8, 131.3, 130.2, 124.5, 115.1, 114.6, 114.4, 74.9, 72.3, 56.5, 55.5; IR (Diamond prism) ν_{max} 3008, 2969, 2896, 2834, 2240, 2163, 2105, 1569, 1511, 1461, 1361, 1241, 1180, 1110, 1072, 1029, 998, 840, 813, 752, 663, 574 cm^{-1}; HRMS-ESI (*m/z*) $[M+Na]^+$ calcd for $C_{18}H_{20}O_5Na$ 339.1208, found 339.1205; mp 66 ˚C

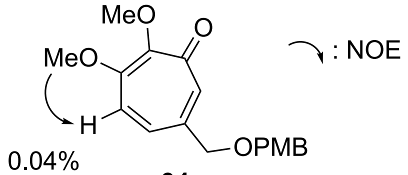

34

*NOE analysis of the compound **35** was unclear to determine the structure in this stage because of the overlapped peaks of methoxy groups. So the structure of **35** was determined after the removal of the PMB group.

7-Hydroxytropolones **37** and **38**

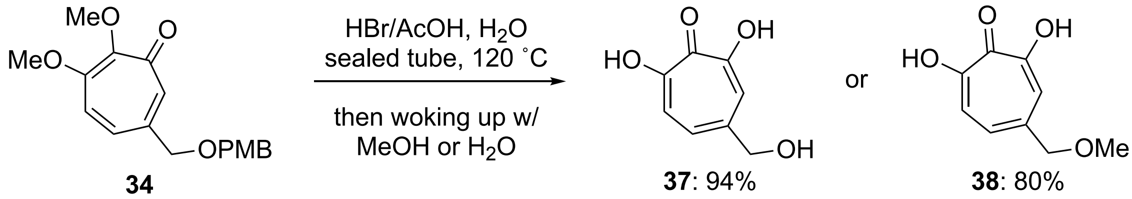

To **34** in a sealed tube was added a 4:1 mixture of 33% HBr/AcOH and H_2O (0.1 M) at room temperature. After being stirred at 120 ˚C for 2 h, the reaction mixture was concentrated under reduced pressure. The residue was purified by Sep-pak® Plus C18 Short Cartridge using acetone/H_2O to afford **37** (6.0 mg scale; 3.0 mg, 94%) as a yellow solid, or using MeOH/H_2O to afford **38** (5.0 mg scale; 2.3 mg, 80%) as a yellow oil.

37: ^1H NMR (500 MHz, CD_3OD) δ 7.73 (s, 1H), 7.60 (d, *J* = 10.9 Hz, 1H), 7.42 (d, *J* = 10.9 Hz, 1H), 4.64 (s, 2H); ^{13}C NMR (125 MHz, CD_3OD) δ 168.9, 161.5, 160.8, 144.5, 127.9, 122.0, 121.2, 67.2; IR (Diamond prism) ν_{max} 3440, 3224, 2545, 2171, 2121, 2086, 1612, 1519, 1454, 1403, 1361, 1276, 1199, 1137, 1091, 1041, 995, 902, 838, 763, 655, 528 cm^{-1}; HRMS-EI (*m/z*) $[M^+]$ calcd for $C_8H_8O_4$ 168.0423, found 168.0426; mp 144

°C (decomp.)

38: ^{1}H NMR (500 MHz, CD_3OD) δ 7.48 (br-s, 1H), 7.41 (d, J = 10.9 Hz, 1H), 7.18 (br-d, J = 10.9 Hz, 1H), 4.42 (s, 2H), 3.40 (s, 3H); ^{13}C NMR (125 MHz, CD_3OD) δ 169.5, 161.4, 161.2, 141.1, 129.0, 121.73, 121.68, 77.7, 58.5; IR (Diamond prism) ν_{max} 3266, 2927, 2823, 1974, 1920, 1859, 1731, 1527, 1419, 1388, 1349, 1272, 1191, 1126, 1087, 1056, 952, 914, 836, 782 cm^{-1}; HRMS-EI (*m/z*) [M$^+$] calcd for $C_9H_{10}O_4$ 182.0579, found 182.0572

Aldehyde **39** and alcohol **S1**

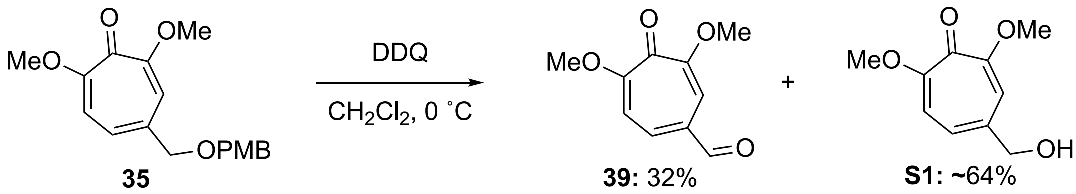

To a solution of **35** (15.1 mg, 47.73 μmol) in CH_2Cl_2 (0.95 mL) was added DDQ (32.5 mg, 0.14 mmol) at 0 °C. After being stirred at 0 °C for 2 h, the reaction mixture was quenched with sat. aq. $NaHCO_3$ (5 mL), extracted with $CHCl_3$:*i*-PrOH = 10:1 (10 mL × 6). The organic layer dried over sodium sulfate, and concentrated under reduced pressure. The residue was purified by preparative TLC ($CHCl_3$/MeOH = 10:1) to afford **39** (4.9 mg, 32%) as a yellow powder. The alcohol **S1** was obtained under the same condition in up to 64% yield as a yellow solid.

39: ^{1}H NMR (500 MHz, $CDCl_3$) δ 9.77 (s, 1H), 7.44 (s, 1H), 7.38 (d, J = 9.7 Hz, 1H), 6.96 (d, J = 9.7 Hz, 1H), 4.03 (s, 3H), 4.02 (s, 3H); ^{13}C NMR (125 MHz, $CDCl_3$) δ 192.0, 174.3, 163.9, 161.0, 135.3, 132.7, 111.3, 107.9, 57.1, 56.8; IR (Diamond prism) ν_{max} 3027, 2962, 2888, 2834, 2387, 2159, 2098, 2005, 1727, 1677, 1608, 1577, 1253, 1226, 1164, 1106, 1006, 979, 890, 829, 767, 674, 597, 547; HRMS-ESI (*m/z*) [M+Na]$^+$ calcd for $C_{10}H_{10}O_4Na$ 217.0477, found 217.0471; mp 159 °C

S1: ^{1}H NMR (500 MHz, $CDCl_3$) δ 7.00 (s, 1H), 6.93 (d, J = 10.3 Hz, 1H), 6.84 (d, J = 10.3 Hz, 1H), 4.62 (s, 2H), 3.94 (s, 3H), 3.92 (s, 3H), 2.36 (br-s, 1H); ^{13}C NMR (125 MHz, $CDCl_3$) δ 173.3, 161.7, 161.4, 139.4, 123.8, 114.7, 114.6, 67.9, 56.62, 56.57; IR (Diamond prism) ν_{max} 3671, 2541, 2225, 2167, 2071, 1997, 1920, 1724, 1589, 1527, 1457, 1346, 1203, 1087, 1052, 995, 941, 763, 694, 497; HRMS-ESI (*m/z*) [M+Na]$^+$ calcd for $C_{10}H_{12}O_4Na$ 219.0633, found 217.0626; mp 98 °C

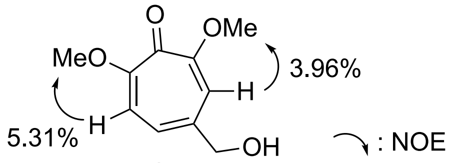

Iso-stipitatic acid (**40**)

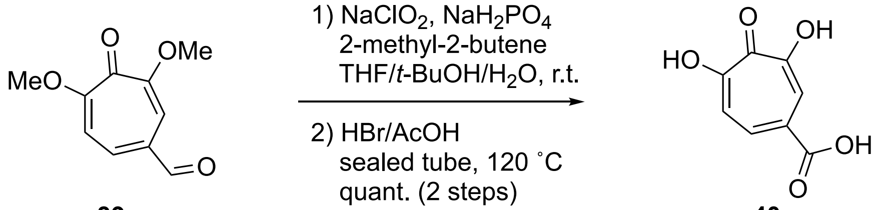

To a solution of **39** (4.0 mg, 20.60 μmol) and 2-methyl-2-butene (43.77 μL, 0.41 mmol) in THF (0.21 mL) and *t*-BuOH (0.21 mL) was added the mixture of $NaClO_2$ (2.8 mg, 30.90 μmol) and NaH_2PO_4 (9.6 mg, 61.80 μmol) in H_2O (0.21 mL) dropwise by a Pasteur pipette at room temperature. After being stirred at room temperature for 30 min, the reaction mixture was directly filtrated by silica gel, eluted with $CHCl_3$/MeOH = 10:1, and concentrated under reduced pressure to afford the crude product as a yellow solid. This crude product was used in the next reaction without further purification.

To the crude product in a sealed tube was added a 4:1 mixture of 33% HBr/AcOH and H_2O (0.42 mL) at room temperature. After being stirred at 120 ˚C for 2 h, the reaction mixture was concentrated under reduced pressure. The residue was purified by Sep-pak® Plus C18 Short Cartridge to afford **40** (3.8 mg, quant. over 2 steps) as a yellow solid.

^{1}H NMR (500 MHz, CD_3OD) δ 8.15 (s, 1H), 8.06 (d, J = 10.9 Hz, 1H), 7.45 (d, J = 10.9 Hz, 1H); ^{13}C NMR (125 MHz, CD_3OD) δ 171.7, 169.2, 164.3, 159.9, 132.3, 130.3, 120.5, 119.5; IR (Diamond prism) ν_{max} 3610, 3556, 3459, 3390, 3178, 2356, 2198, 2156, 2028, 1974, 1685, 1619, 1527, 1365, 1290, 1156, 1023, 953, 843 cm^{-1}; HRMS-ESI (*m/z*) [M–H]$^-$ calcd for $C_8H_5O_5$ 181.0137, found 181.0128; mp 170 ˚C (decomp.)

2-3. Synthesis of 6,7-dihydroxytropolones

Alcohol **22**

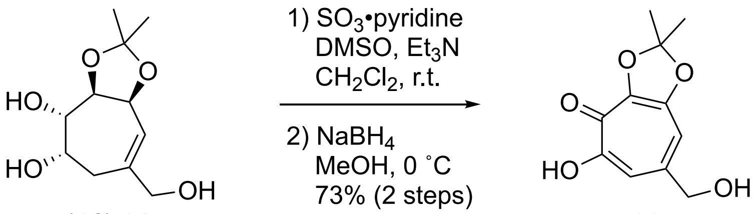

(*6S*)-**21** **22**

To a solution of (*6S*)-**21** (20.3 mg, 88.16 μmol) in CH_2Cl_2 (0.88 mL) was added DMSO (93 μL, 1.32 mmol), Et_3N (0.37 mL, 2.64 mmol), and SO_3•pyridine (0.14 g, 0.88 mmol) at 0 ˚C. The mixture was stirred at room temperature for 8 h, then quenched with 1M HCl (10 mL), and extracted with $CHCl_3$ (10 mL × 3). The combined organic layer was dried over sodium sulfate, and concentrated under reduced pressure to yield crude product as a dark brown amorphous. This crude product was used in the next reaction without further purification.

To a solution of the crude product in MeOH (0.88 mL) was added $NaBH_4$ (3.3 mg, 88.16 μmol) at 0 ˚C. After being stirred at 0 ˚C for 5 min, the reaction mixture was quenched with acetone (1 mL). The resulting mixture was concentrated under reduced pressure. The residue was purified by Sep-pak® Plus C18 Short Cartridge to afford **22** (14.4 mg, 73% over 2 steps) as a yellow solid.

^{1}H NMR (500 MHz, CD_3OD) δ 7.18 (s, 1H), 7.00 (s, 1H), 4.54 (s, 2H), 1.76 (s, 6H); ^{13}C NMR (125 MHz, CD_3OD) δ 168.9, 159.3, 156.5, 150.4, 146.8, 119.7, 116.7, 110.0, 66.7, 26.0; IR (Diamond prism) ν_{max} 3309, 3201, 2923, 2857, 2071, 2017, 1589, 1531, 1488, 1442, 1380, 1311, 1187, 1126, 1052, 979, 840, 767, 717 cm^{-1}; HRMS-ESI (*m/z*) $[M+Na]^+$ calcd for $C_{11}H_{12}O_5Na$ 247.0582, found 247.0577; mp 168 ˚C

6,7-Dihydroxytropolones **42** and **43**

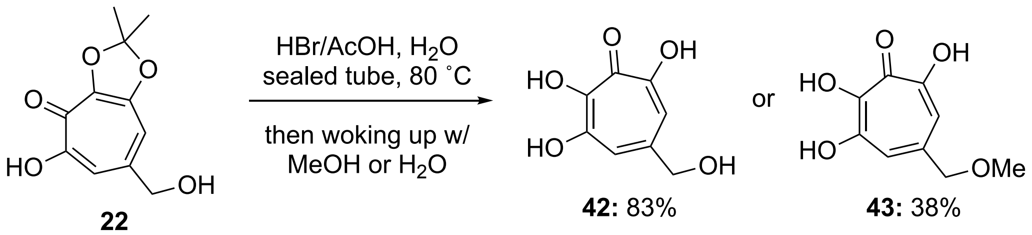

To **22** in a sealed tube was added a 4:1 mixture of 33% HBr/AcOH and H_2O (0.1 M) at room temperature. After being stirred at 80 ˚C for 2 d, the reaction mixture was concentrated under reduced pressure. The residue was purified by Sep-pak® Plus C18 Short Cartridge using acetone/H_2O to afford **42** (14.4 mg scale; 9.8 mg, 83%) as a yellow solid, or using MeOH/H_2O to afford **43** (5.1 mg scale; 1.7 mg, 38%) as a yellow oil.

42: ^{1}H NMR (500 MHz, CD_3OD) δ 7.15 (s, 2H), 4.52 (s, 2H); ^{13}C NMR (125 MHz, CD_3OD) δ 158.4, 157.2, 143.8, 117.2, 67.3; IR (Diamond prism) ν_{max} 3475, 3205, 2915, 2861, 2229, 2179, 2098, 1997, 1940, 1589, 1523, 1488, 1384, 1334, 1234, 1187, 1095, 1052, 979, 725, 659, 555 cm^{-1}; HRMS-EI (*m/z*) [M^+] calcd for $C_8H_8O_5$ 184.0372, found 184.0388; mp 162 ˚C

43: ^{1}H NMR (500 MHz, CD_3OD) δ 7.10 (s, 2H), 4.38 (s, 2H), 3.40 (s, 3H); ^{13}C NMR (125 MHz, CD_3OD) δ 158.4, 157.2, 140.1, 117.8, 77.6, 58.3; IR (Diamond prism) ν_{max} 3424, 3158, 2923, 2202, 2159, 2028, 1986, 1940, 1893, 1573, 1454, 1396, 1357, 1288, 1234, 1191, 1083, 1037, 983, 917, 717, 632 cm^{-1}; HRMS-ESI (*m/z*) $[M–H]^-$ calcd for $C_9H_9O_5$ 197.0450, found 197.0450; mp 120 ˚C

Azide **44**

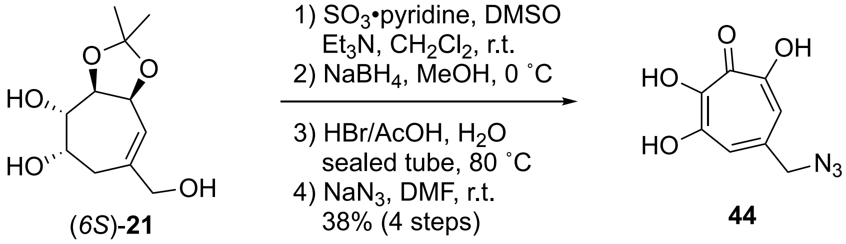

According to the procedure for preparation of **22**, Parikh-Doering oxidation of (*6S*)-**21** (21.4 mg, 92.93 µmol) and subsequent reduction of the resulting aldehyde afforded the crude product as a dark brown amorphous. This crude material was used in

subsequent reaction without further purification.

To crude **22** in a sealed tube was added a 4:1 mixture of 33% HBr/AcOH and H_2O (0.93 mL) at room temperature. After being stirred at 80 ˚C for 2 d, the reaction mixture was concentrated under reduced pressure to yield the crude product.

To a solution of the crude product in DMF (0.93 mL) was added NaN_3 (18.1 mg, 0.28 mmol) at room temperature. After being stirred at room temperature for 2.5 h, the reaction mixture was quenched with H_2O (10 mL). The organic layer was separated, and the aqueous layer was extracted with EtOAc (10 mL × 3). The combined organic layer was dried over sodium sulfate, and concentrated under reduced pressure. The residue was purified by Sep-pak® Plus C18 Short Cartridge to afford **44** (7.4 mg, 38% over 4 steps) as a yellow solid.

^{1}H NMR (500 MHz, CD_3OD) δ 7.09 (s, 2H), 4.35 (s, 2H); ^{13}C NMR (125 MHz, CD_3OD) δ 158.0, 157.6, 137.3, 118.8, 58.8; IR (Diamond prism) ν_{max} 3486, 3278, 3151, 3050, 2260, 2183, 2094, 1978, 1940, 1878, 1592, 1515, 1481, 1396, 1330, 1191, 1095, 1025, 863, 775, 732 cm^{-1}; HRMS-ESI (*m/z*) $[M–H]^-$ calcd for $C_8H_6O_4N_3$ 208.0358, found 208.0358; mp 159 ˚C

2-4. Data comparison of synthetic viticolin A with the natural product

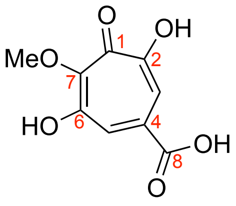

viticlin A

	Synthetic viticolin A[a]		Reported[b]	
	^{13}C NMR	^{1}H NMR	^{13}C NMR	^{1}H NMR
Position	δ_C (ppm)	δ_H (ppm), [mult., Int.]	δ_C (ppm)	δ_H (ppm), [mult., Int.]
1	172.0		170.5	
2	163.3		163.8	
3	112.0	7.72 (s, 1H)	111.0	7.72 (s, 1H)
4	134.4		133.0	
5	123.4	7.82 (s, 1H)	122.5	7.83 (s, 1H)
6	160.6		161.0	
7	150.0		152.0	
7-OMe	59.6	3.95 (s, 3H)	58.8	3.94 (s, 3H)
8	168.7		167.0	

[a]exp. ^{13}C NMR: 125 MHz, ^{1}H NMR: 500 MHz in $(CD_3)_2CO$

[b]exp. ^{13}C NMR: 100 MHz, ^{1}H NMR: 400 MHz in $(CD_3)_2CO$

*Chemical sifts of ^{13}C NMR were somewhat different from the reported one probably because of the purity of the natural product. Spectra data of the synthetic viticolin A were producibly obtained in the reasonable way.

^{1}H NMR

synthetic (500 MHz, CD_3OD)

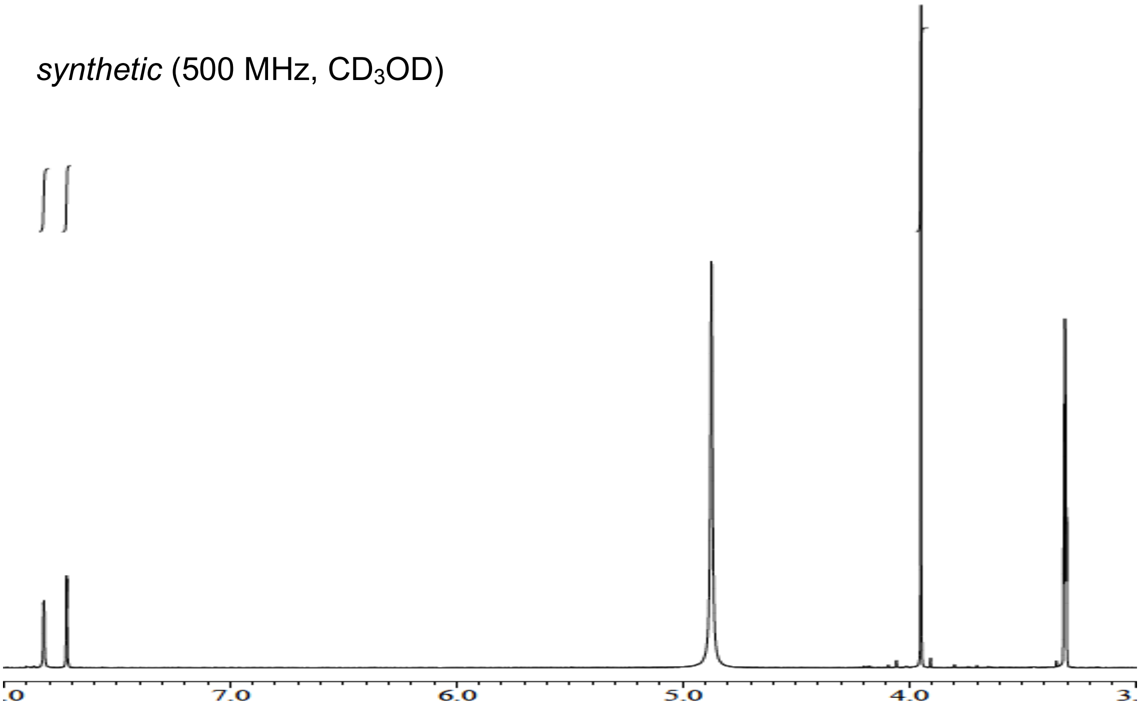

natural (400 MHz, CD_3OD)

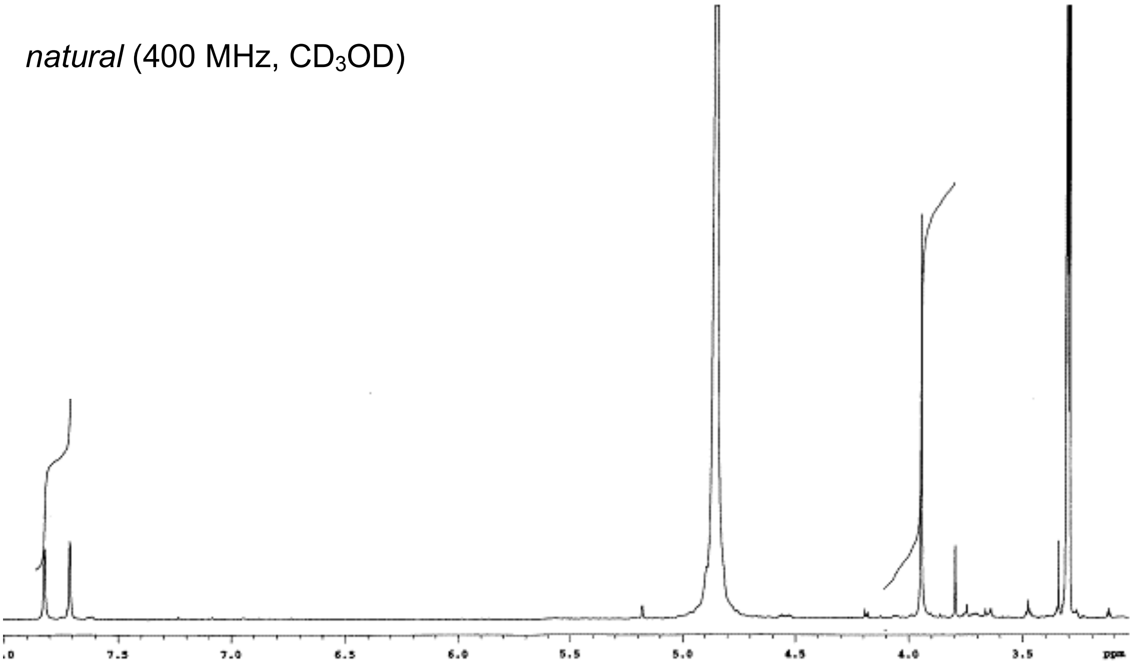

^{13}C NMR

synthetic (125 MHz, CD_3OD)

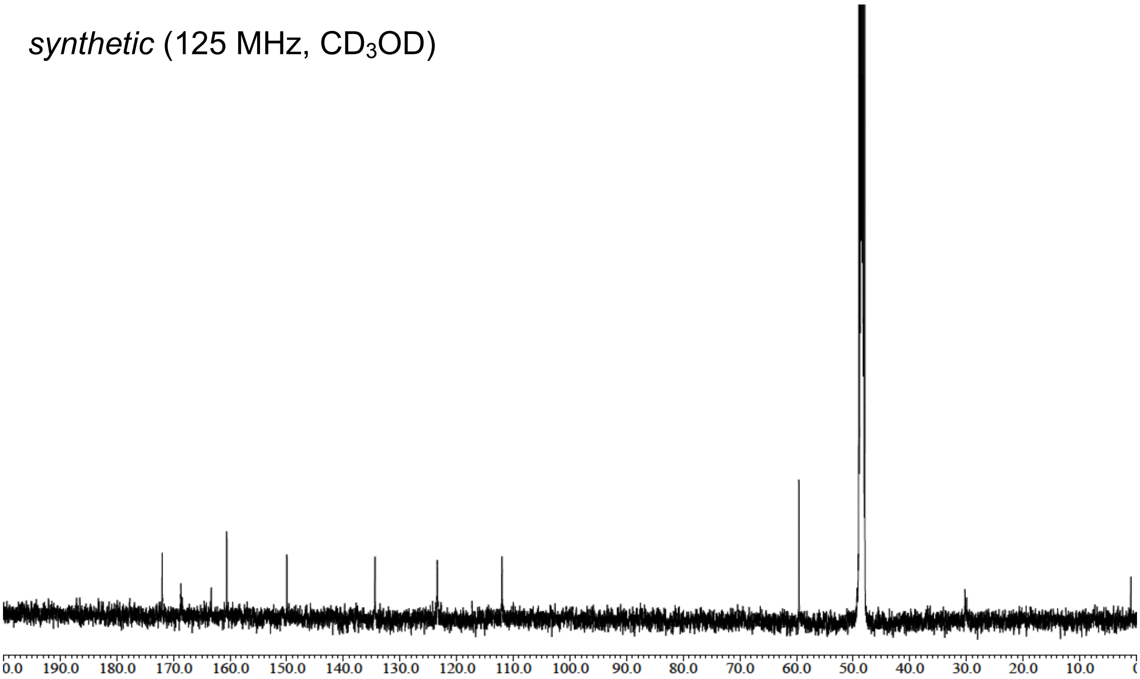

natural (100 MHz, CD_3OD)

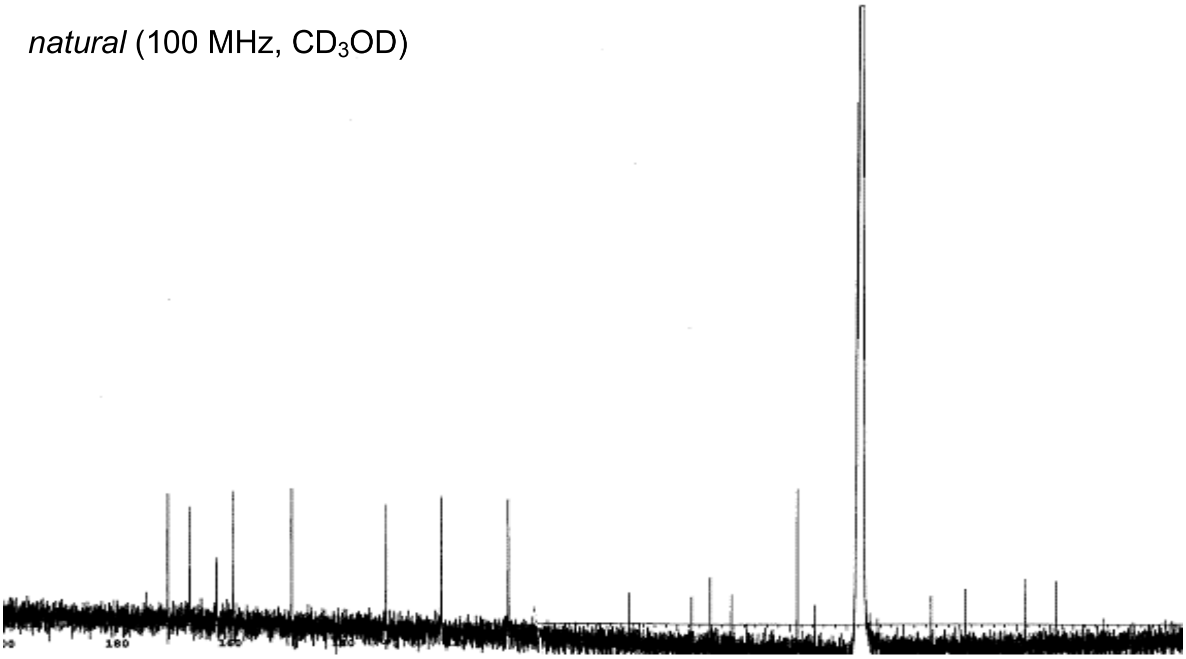

3. Biological evaluation of synthesized derivatives

3-1. *In vitro* cultivation of *Plasmodium falciparum* and the antimalarial assay

In vitro cultivation and antimalarial activity against the *Plasmodium falciparum* K1 (chloroquine-resistant) parasite strain were measured using the method described previously[1]. Briefly, the *P. falciparum* K1 strain was cultured in human erythrocytes in RPMI medium supplemented with 10% human plasma at 37 ˚C, under 93% N_2, 4% CO_2, and 3% O_2. Asynchronous parasites (2% hematocrit and 0.5 or 1% parasitaemia) were seeded in a 96-well microtiter plate and serially diluted test compounds were added. Positive controls, such as chloroquine and artemisinin, were added in a similar fashion. After 72-hours incubation, parasite lactate dehydrogenase (p-LDH) was assayed using a slight modification of the procedure reported by Makler *et al*[2] and Vivas *et al*[3]. The 50% inhibitory concentration (IC_{50}) value was calculated from a dose response curve. This study was approved by "Kitasato Institute Hospital Research Ethics Committee (No12102)" using human erythrocytes donated by volunteers.

3-2. Cytotoxic assay against MRC-5 cells

Measurement of cytotoxicity against human fetal lung fibroblast MRC-5 cells was carried out as described previously[4].

1) K. Otoguro, A. Kohana, C. Manabe, A. Ishiyama, H. Ui, K. Shiomi, H. Yamada, S. Ōmura, *J. Antibiot.* **2001**, *54*, 658-663. "Potent antimalarial activities of polyether antibiotic, X-206."
2) M. T. Makler, J. M. Ries, J. A. Williams, J. E. Bancroft, R. C. Piper, B. L. Gibbins, D. J. Hinrichs, *Am. J. Med. Hyg.* **1993**, *48*, 739-741. "Parasite lactate dehydrogenase as an assay for *Plasmodium falciparum* drug sensitivity."
3) L. Vivas, A. Easton, H. Kendrick, A. Cameron, J.-L. Lavandera, D. Barros, F. G. de las Heras, R. L. Brady, S. L. Croft, *Exper. Parasitol.* **2005**, *111*, 105-114. "*Plasmodium falciparum*: Stage specific effects of a selective inhibitor of lactate dehydrogenase."
4) K. Otoguro, H. Ui, A. Ishiyama, N. Arai, M. Kobayashi, Y. Takahashi, R. Masuma, K. Shiomi, H. Yamada, S. Ōmura, *J. Antibiot.* **2003**, *56*, 322-324. "*In vitro* antimalarial activities of the microbial metabolites."

Table S1. *In vitro* and *in vivo* antimalarial activity and cytotoxicity of troponoids

Compound	IC_{50} (μg/mL)		Selectivity index MRC-5/K1
	Antimalarial activity (K1 strain)	Cytotoxicity (MRC-5)	
**1** (natural)	0.01	57.20	5720
**1** (synthetic)	0.0087	4.18	480.46
**2**	7.07	>100	>14
**3**	9.92	>100	>10
**4**	0.84	6.79	8.08
**5**	>12.50	-	-
**6**	2.74	-	-
**7**	0.89	0.24	0.27
**8**	4.92	5.80	1.20
**23**	>12.50	-	-
**24**	>12.50	-	-
**25**	>12.50	-	-
**26**	>12.50	-	-
**27**	>12.50	-	-
**28**	>12.50	-	-
**29**	0.073	11.85	162.33
**37**	0.728	0.00057	0.0008
**38**	0.380	0.00024	0.0006
**40**	0.387	18.65	47.82
**42**	0.038	0.00435	0.115
**43**	0.044	0.00181	0.041
**44**	0.096	0.00073	0.012
**chloroquine**	0.18	18.60	103
**artemisinin**	0.0057	45.17	7.925

K1: chloroquine-resistant

MRC-5: human diploid embryonic cell line

4. Purity assessment of evaluated derivatives

Purity measurement of the tested compounds was carried out by LC-UV analysis, performed on the Agilent 1100 system (Agilent Technology, Inc.) under the following condition; column, Symmetry C18 (Waters Co., Ltd., 2.1 φ × 150 mm); UV detection, 210 and 254 nm; flow rate, 0.2 mL/min; mobile phase, MeCN-H_2O with 0.05% H_3PO_4, (5-100% linear gradient over 20 min).

Compound	Retention time (min)	Peak area
**1**	5.64	97%
**29**	6.14	98%
**37**	4.71	97%
**38**	10.17	97%
**40**	7.94	99%
**42**	3.21	>99%
**43**	3.22	97%
**44**	10.55	99%

5. Reaction screenings

Table S2. Multi-oxidation of tetraol 18

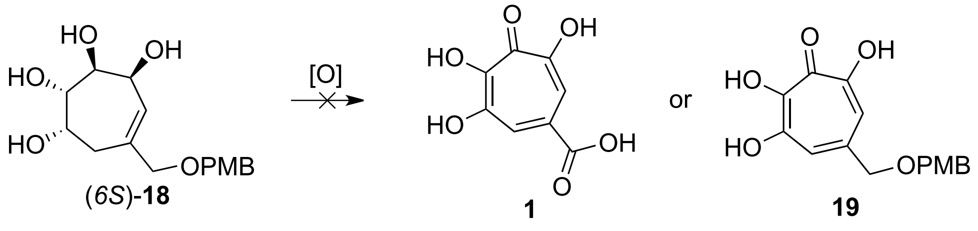

Entry	Condition	Result
1	Jones reagent, acetone, 0 ˚C	Decomp.
2	PCC, Celite®, CH_2Cl_2, r.t.	Decomp.
3	DDQ, CH_2Cl_2, 0 ˚C to r.t.	Complex mixture
4	CAN, MeCN/H_2O, r.t.	Complex mixture
5	$(COCl)_2$, DMSO, CH_2Cl_2, then Et_3N, –78 ˚C	**S2** was observed.
6	SO_3•pyridine, DMSO, Et_3N, CH_2Cl_2, r.t.	**S2** was observed.
7	IBX, DMSO, r.t.	Decomp.
8	DMP, CH_2Cl_2, r.t.	Decomp.
9 [a)]	**S3**, Oxone®, MeCN then H_2O, 70 ˚C	Decomp.

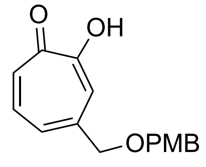

S2

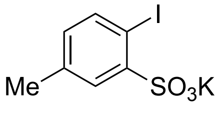

S3: *pre*-MIBSK

a) M. Uyanik, M. Akakura, K. Ishihara, *J. Am. Chem. Soc.* **2009**, *131*, 251-262.

Table S3. Stepwise oxidation of diol 17

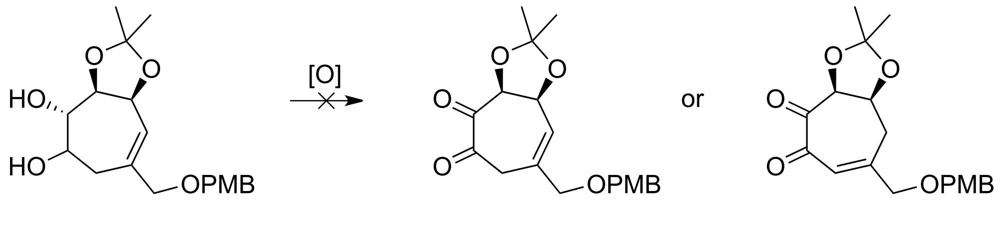

Entry	Condition	Result
1	PDC, Celite®, CH_2Cl_2, r.t.	Decomp.
2	TPAP, NMO, MS4A, CH_2Cl_2, r.t.	Complex mixture
3	$Al(OPr)_3$, acetone, r.t.	Recovery of SM
4[b)]	EtMgBr, **S4**, THF, 0 ˚C to r.t.	No reaction
5	DMP, CH_2Cl_2, r.t.	Decomp.
6	IBX, DMSO, r.t.	Decomp.
7[c)]	**S5**, DBU, CH_2Cl_2, –78 ˚C to r.t.	Complex mixture
8	SO_3•pyridine, DMSO, Et_3N, CH_2Cl_2, 0 ˚C to r.t.	Complex mixture
9	$(COCl)_2$, DMSO, CH_2Cl_2, then Et_3N, –78 ˚C	Complex mixture

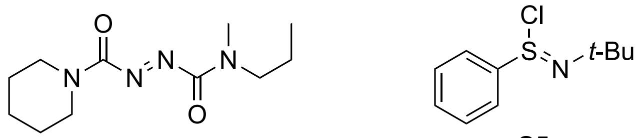

b) K. Narasaka, A. Morikawa, K. Saigo, T. Mukaiyama, *Bull. Chem. Soc. Jpn.* **1977**, *50*, 2773-2776.

c) T. Mukaiyama, J. Matsuo, M. Yanagisawa, *Chem. Lett.* **2000**, *29*, 1072-1073.

Table S4. Multi-oxidation of triol 21

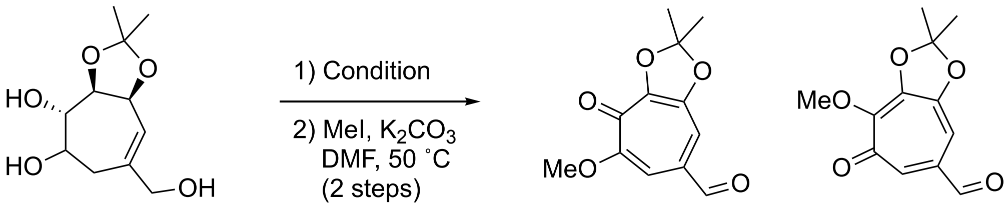

Entry	Condition	Result
1	IBX, DMSO, r.t.	Decomp.
2	$(COCl)_2$, DMSO, CH_2Cl_2, then Et_3N, –78 ˚C	Complex mixture
3	SO_3•pyridine, DMSO, Et_3N, CH_2Cl_2, 0 ˚C to r.t.	**S6 + S6': 28% (1:1)**
4	AZADO, $PhI(OAc)_2$, CH_2Cl_2/H_2O, r.t.	Decomp.
9[a)]	**S3**, Oxone®, MeCN then H_2O, 70 ˚C	Decomp.

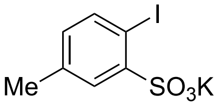

S3: *pre*-MIBSK

6. Spectra Charts

Carboxylic acid **27**

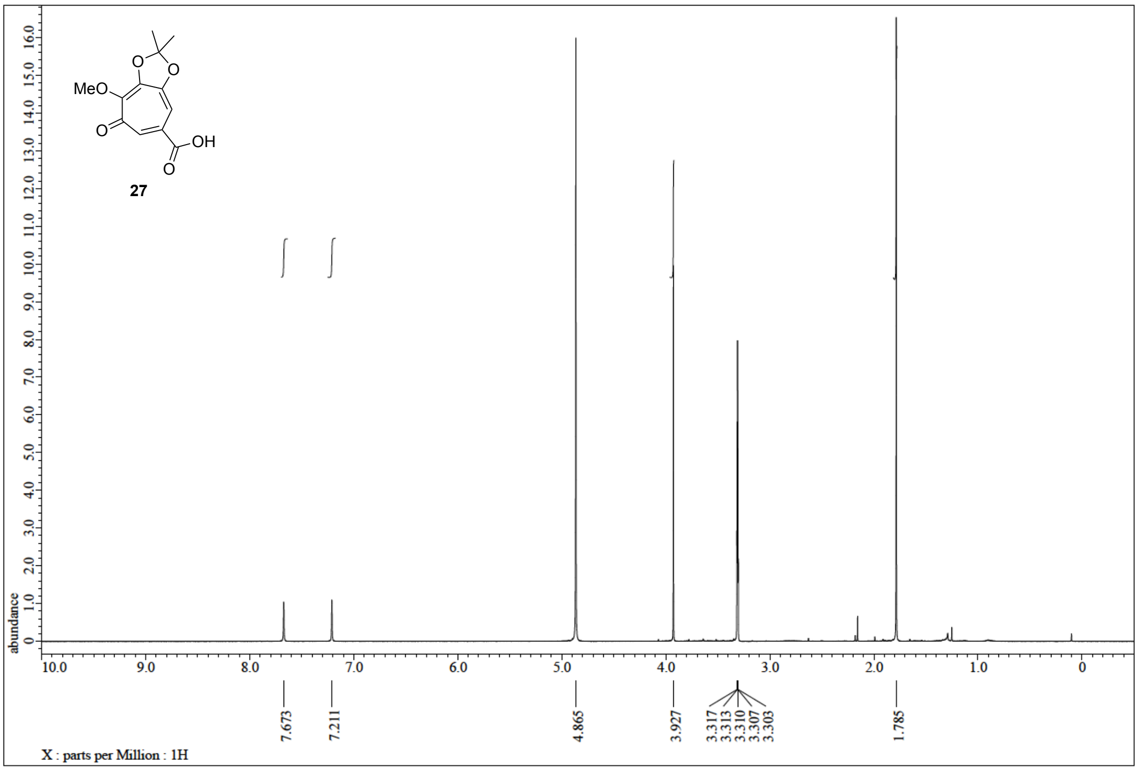

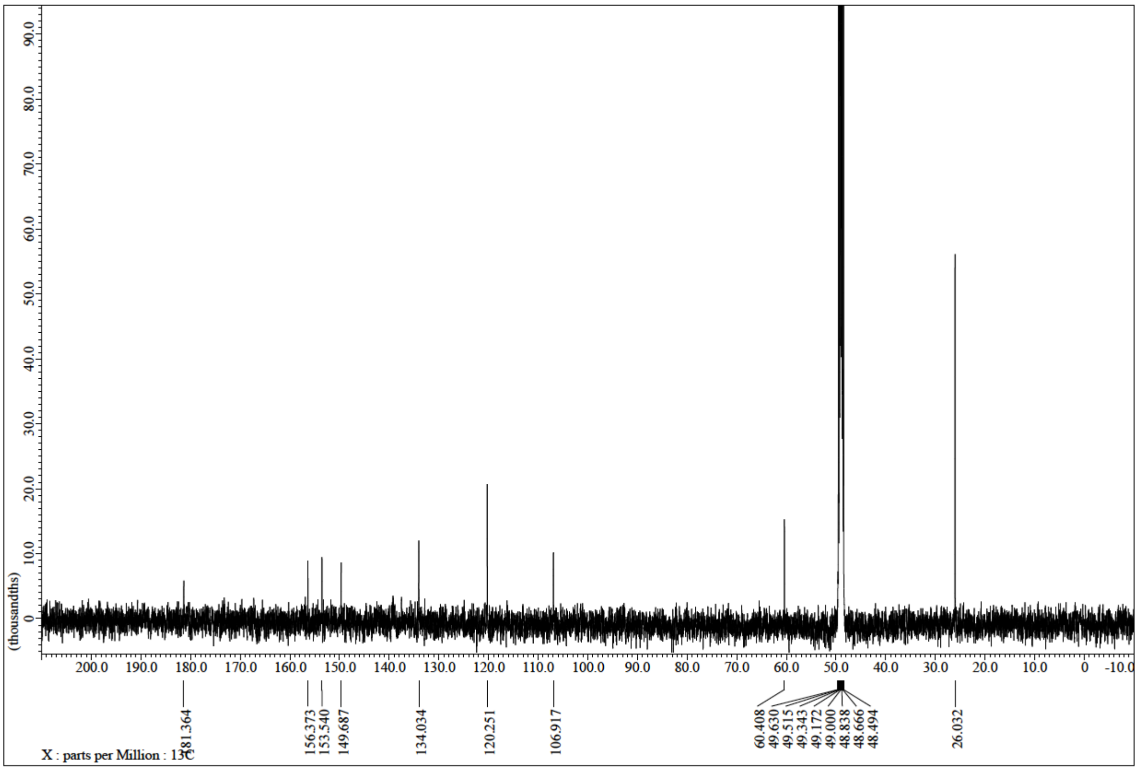

Carboxylic acid **28**

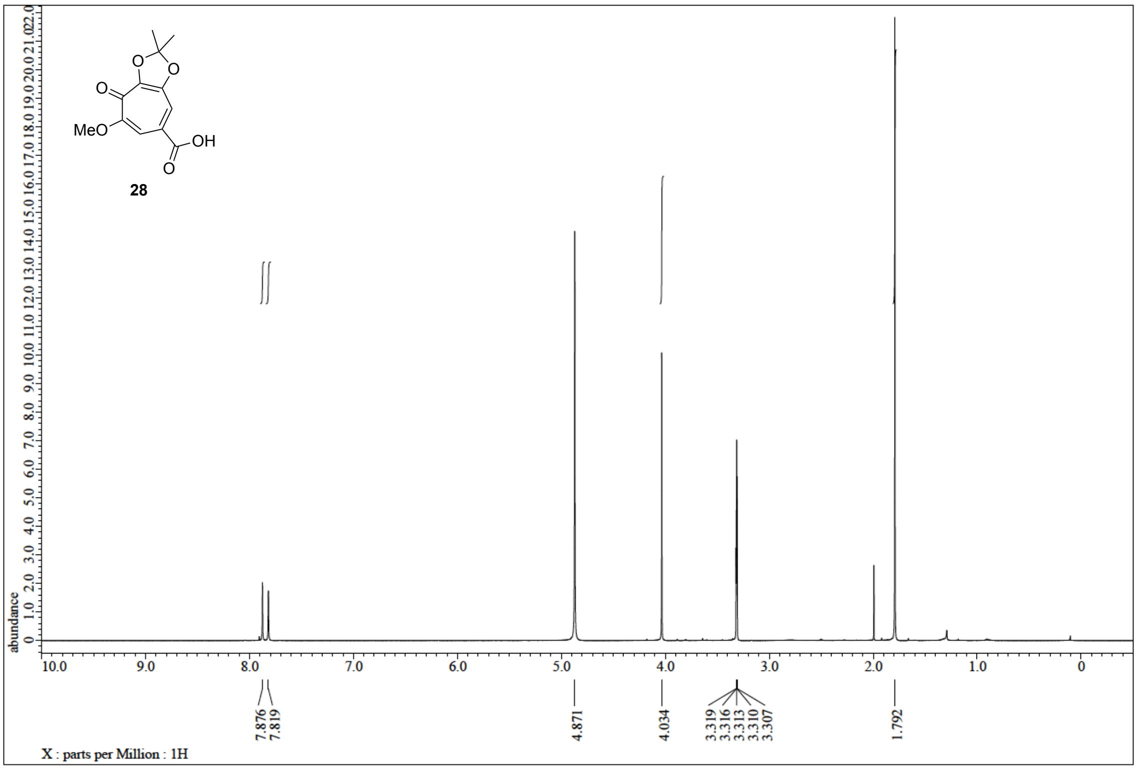

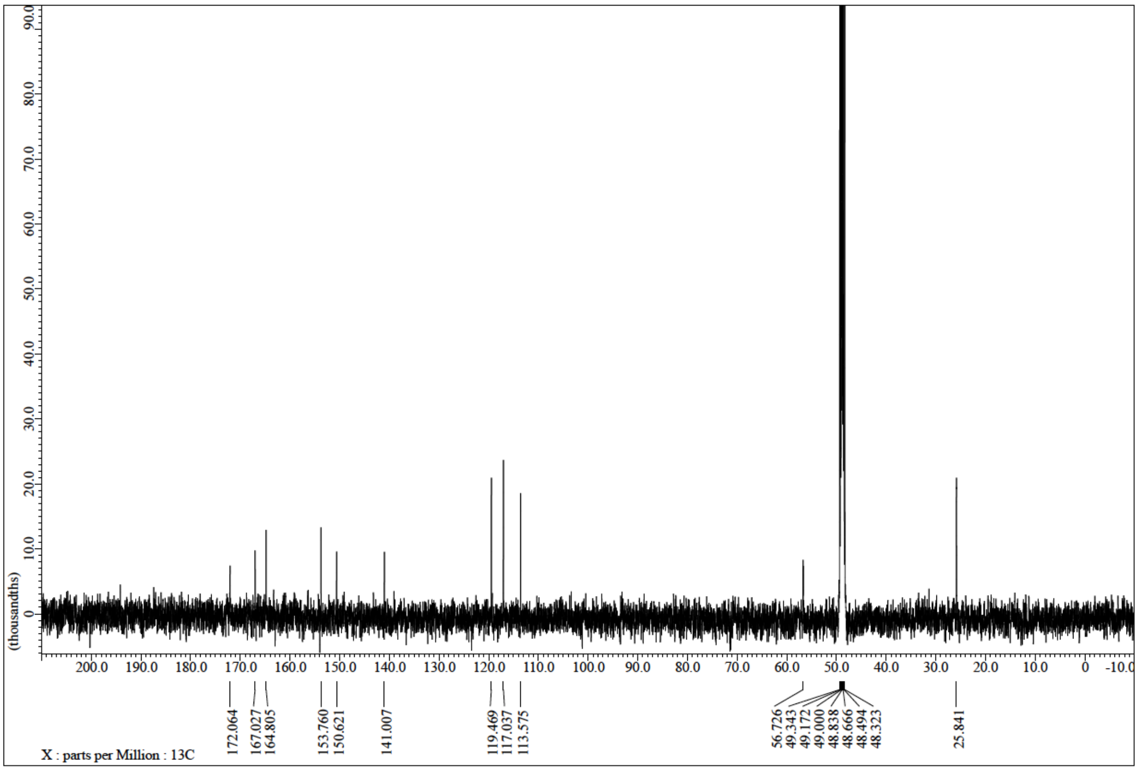

Iso-viticolin A (**29**)

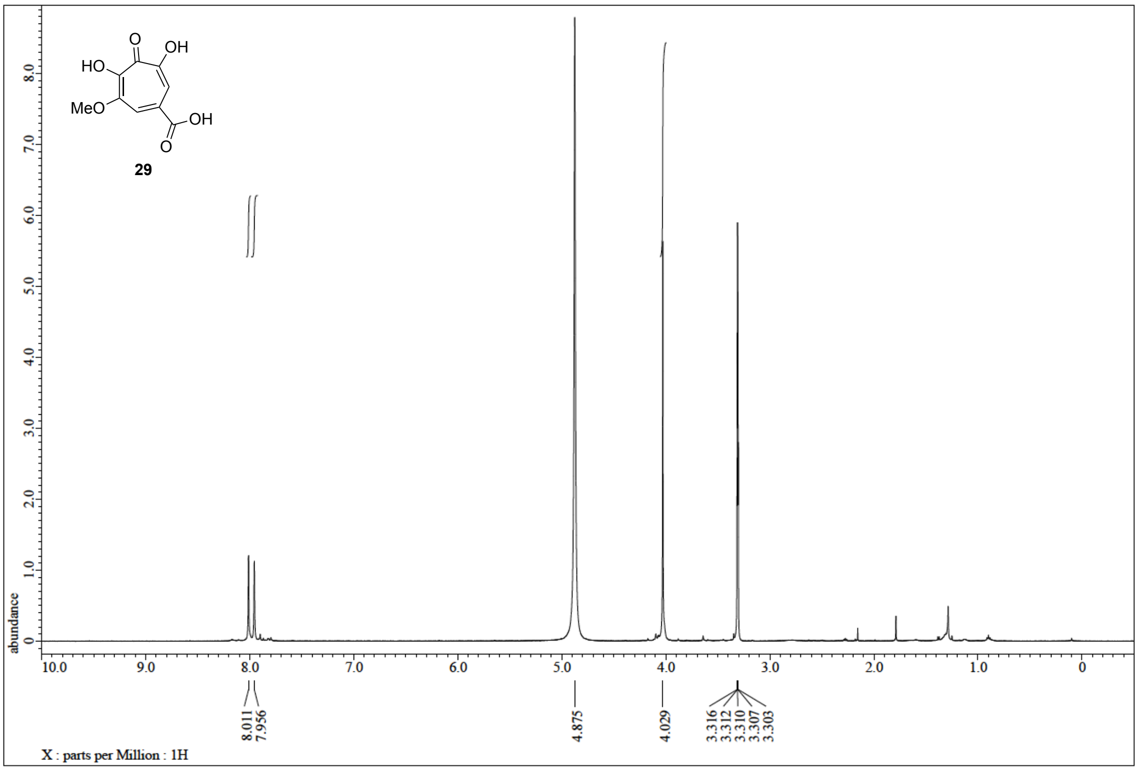

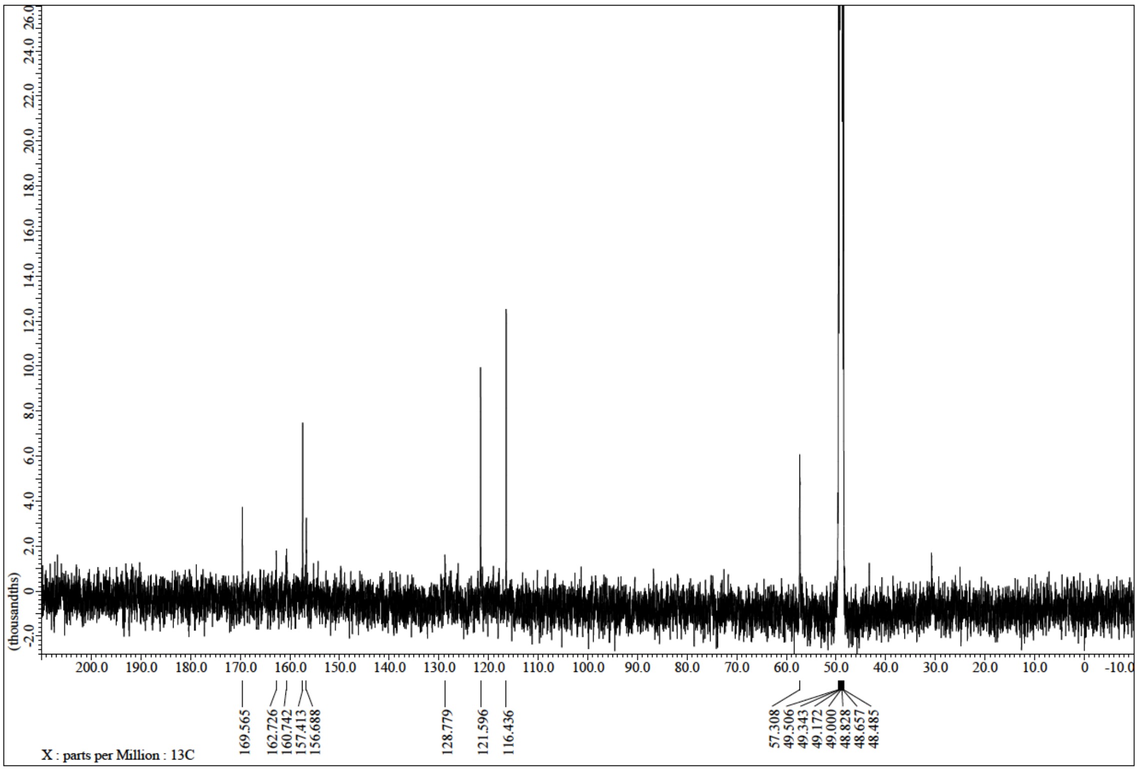

Methylether **30**

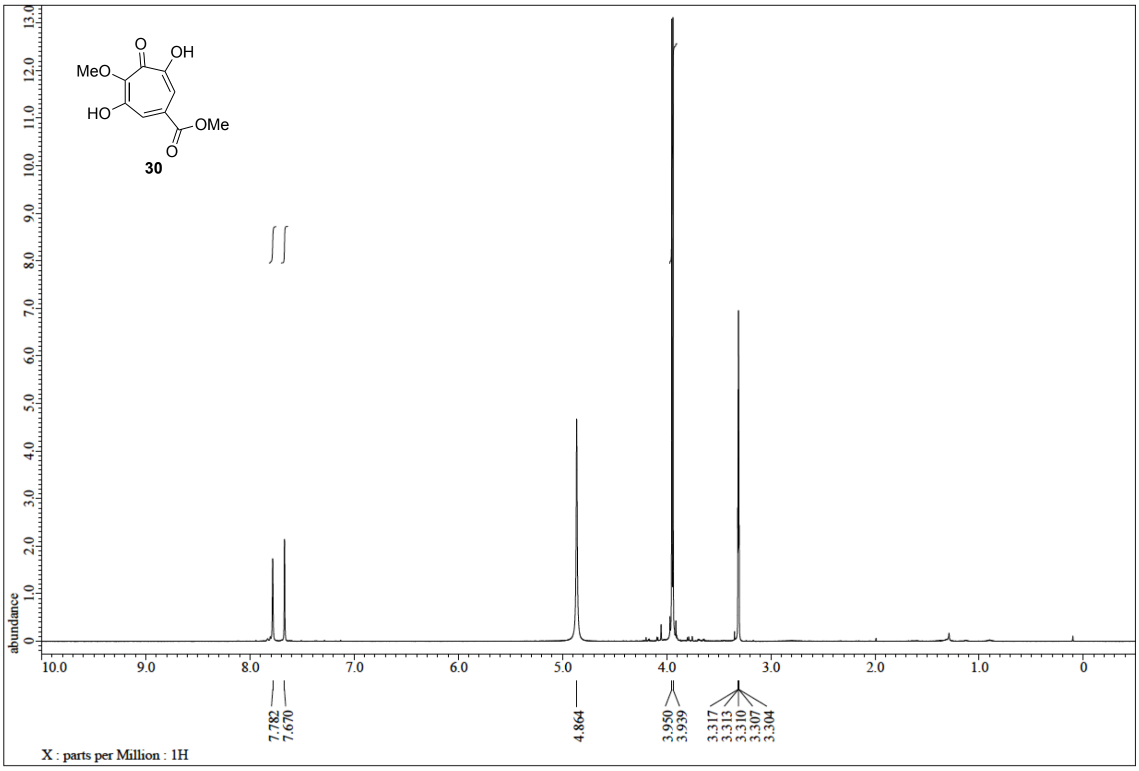

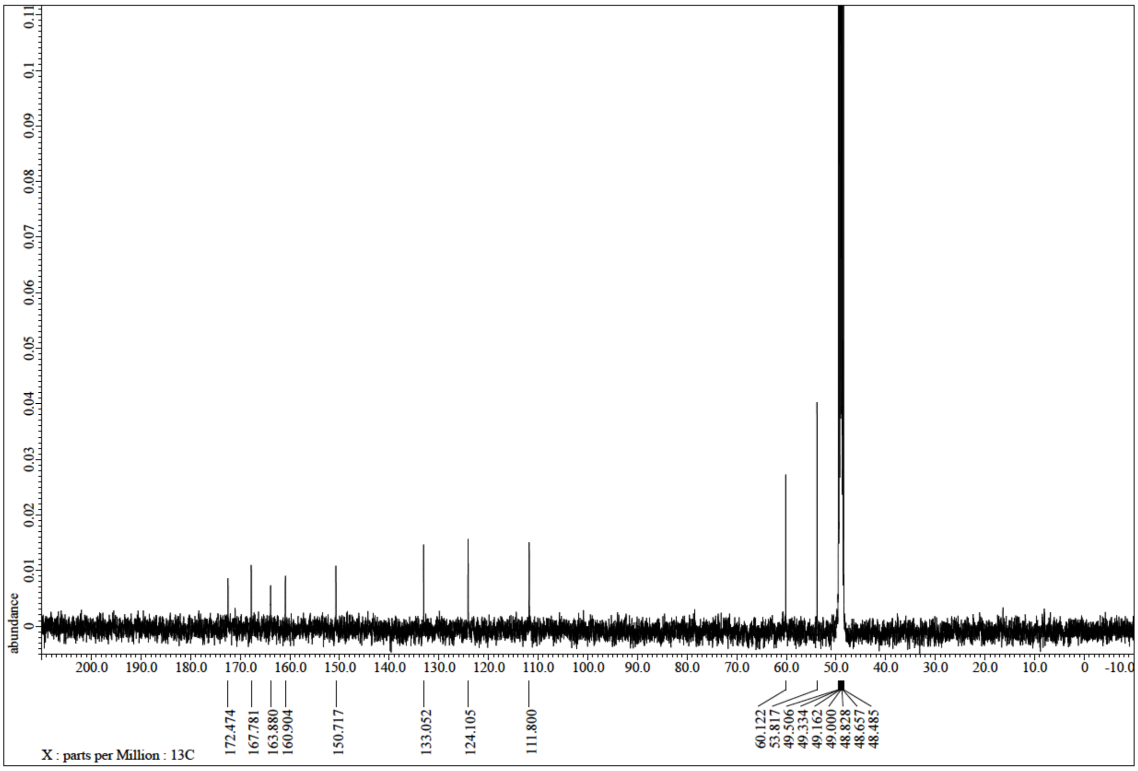

Viticolin A (**3**)

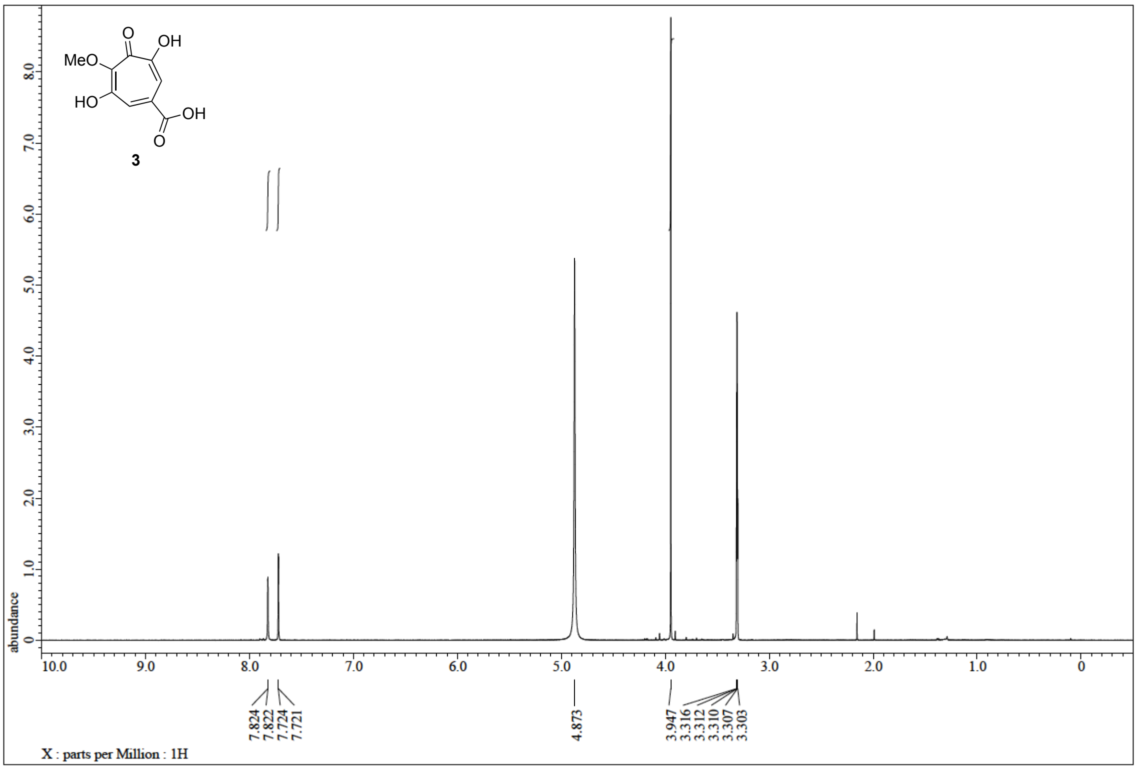

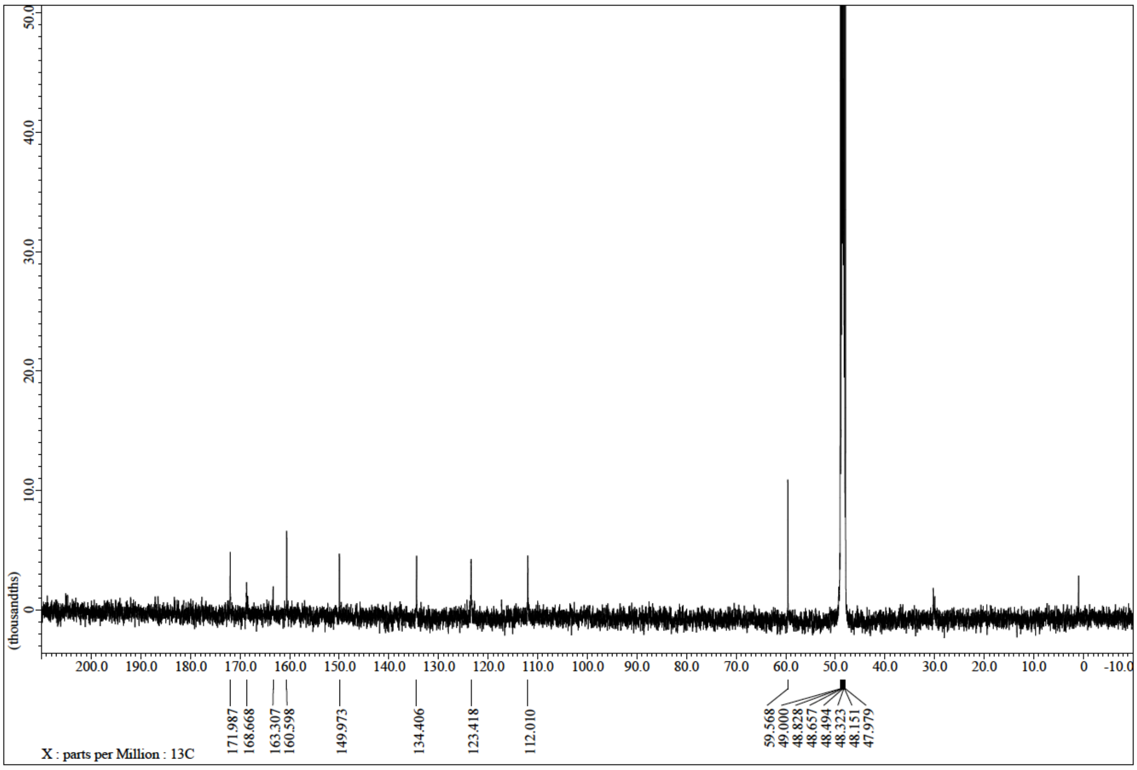

Diol (*6S*)-**31**

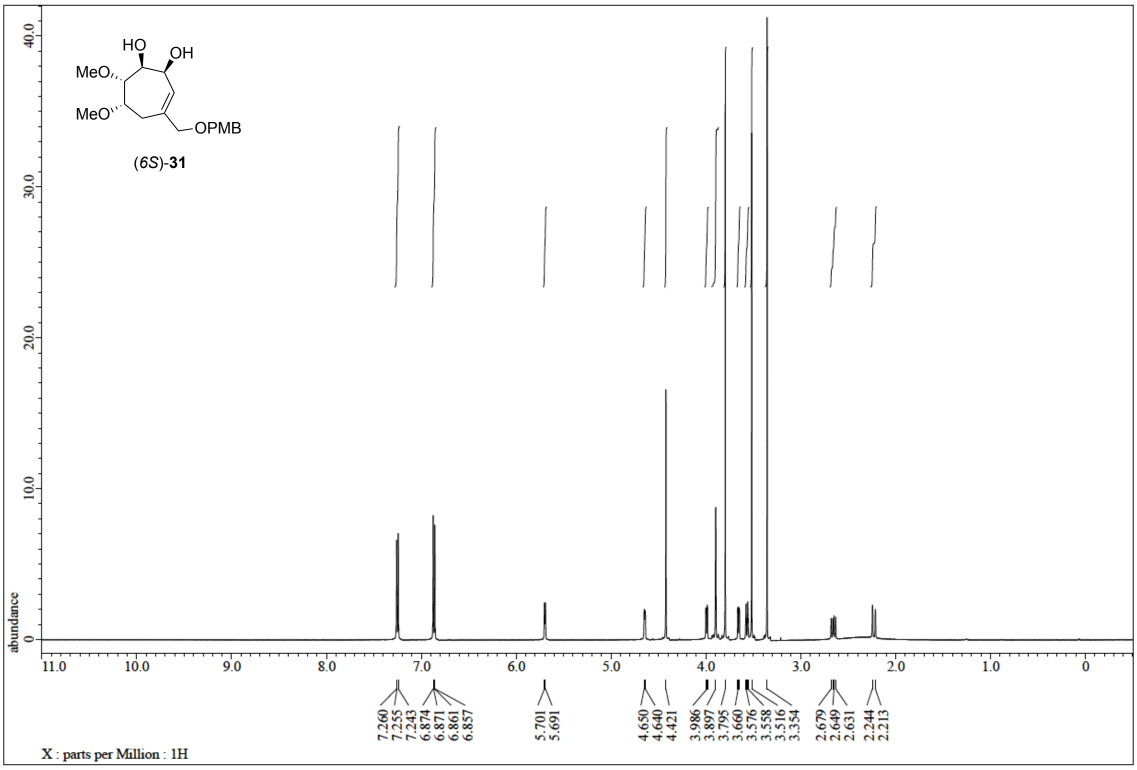
HO
OH
MeO
MeO
OPMB
(6S)-31
abundance
X : parts per Million : 1H
7.260
7.255
7.243
6.874
6.871
6.861
6.857
5.701
5.691
4.650
4.640
4.421
3.986
3.897
3.795
3.660
3.576
3.558
3.516
3.354
2.679
2.649
2.631
2.244
2.213

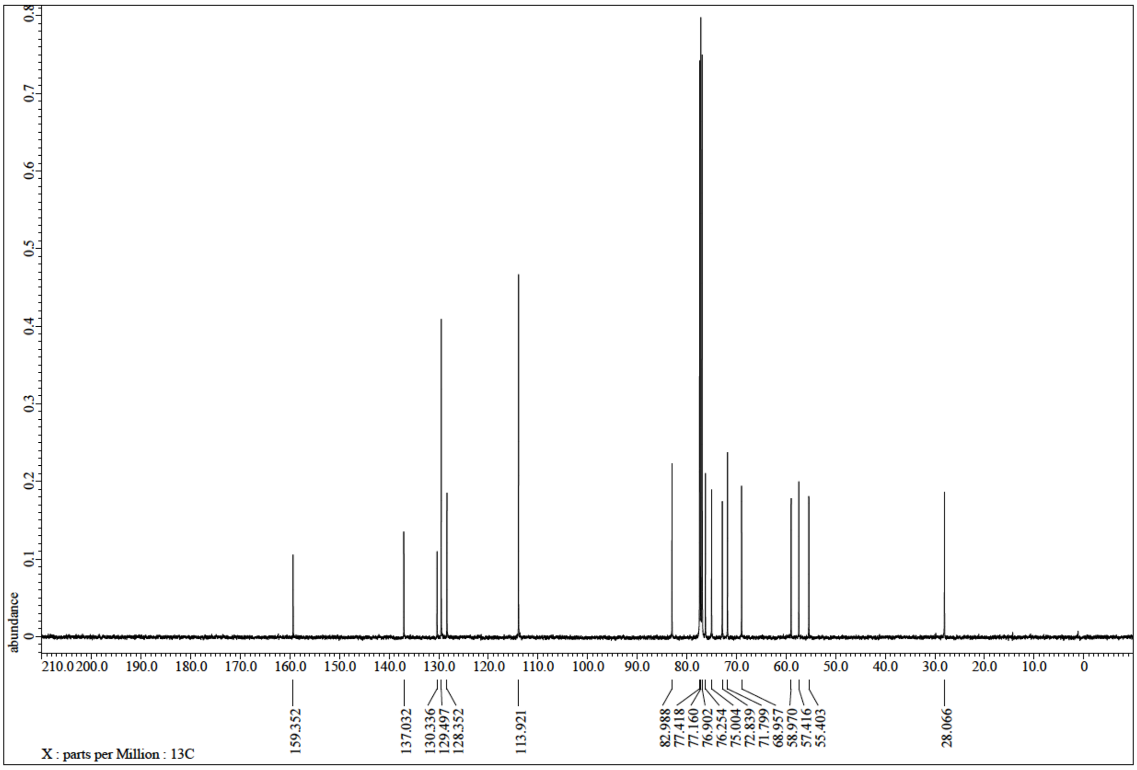
abundance
X : parts per Million : 13C
159.352
137.032
130.336
129.497
128.352
113.921
82.988
77.418
77.160
76.902
76.254
75.004
72.839
71.799
68.957
58.970
57.416
55.403
28.066

Tropone **34**

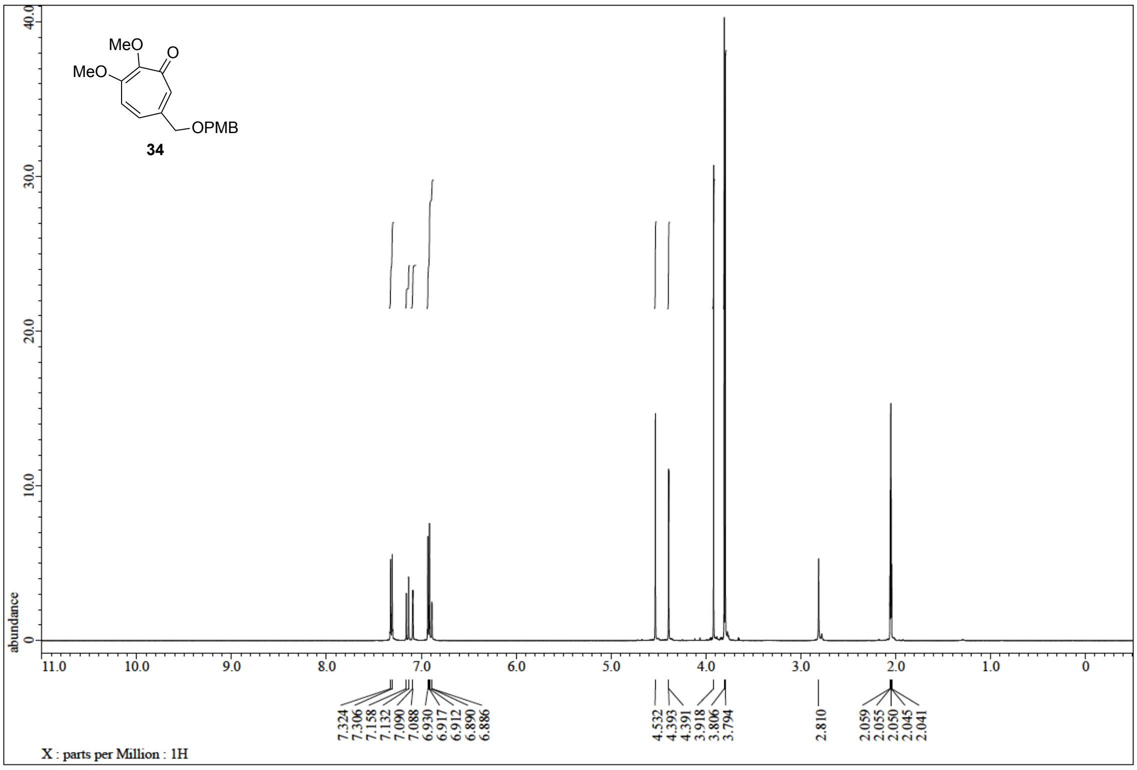
MeO
O
MeO
OPMB
34
abundance
X : parts per Million : 1H

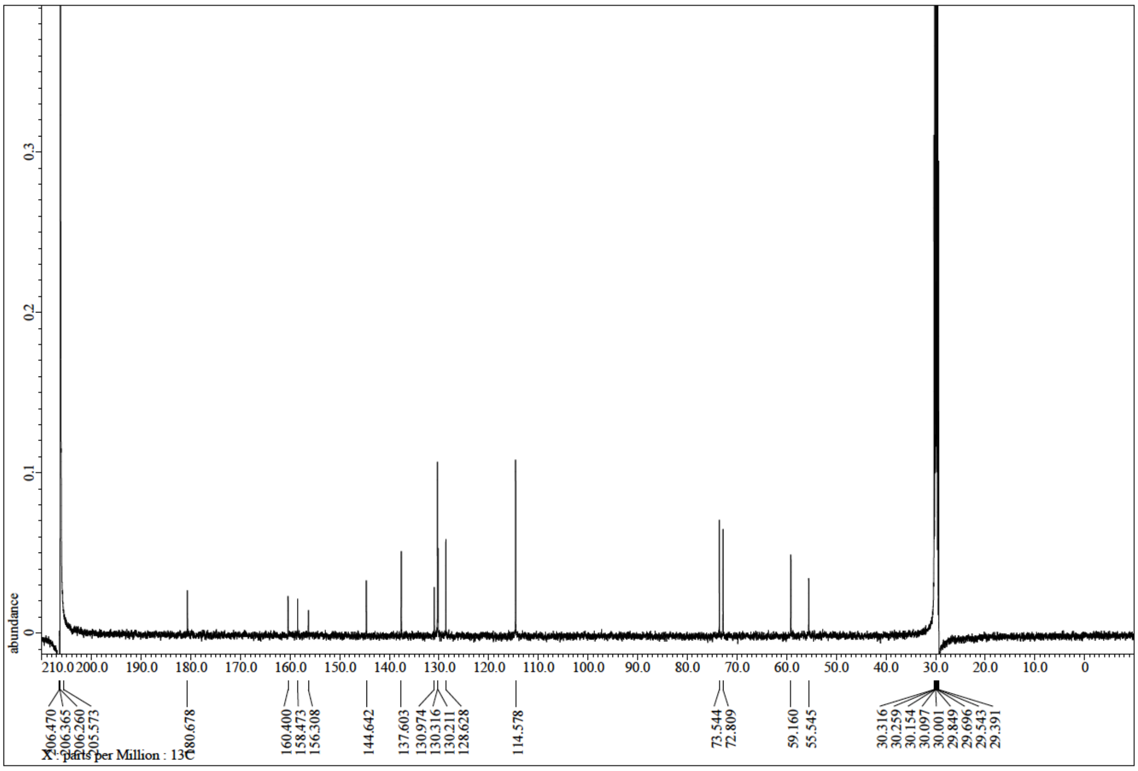
abundance
X : parts per Million : 13C

Tropone **35**

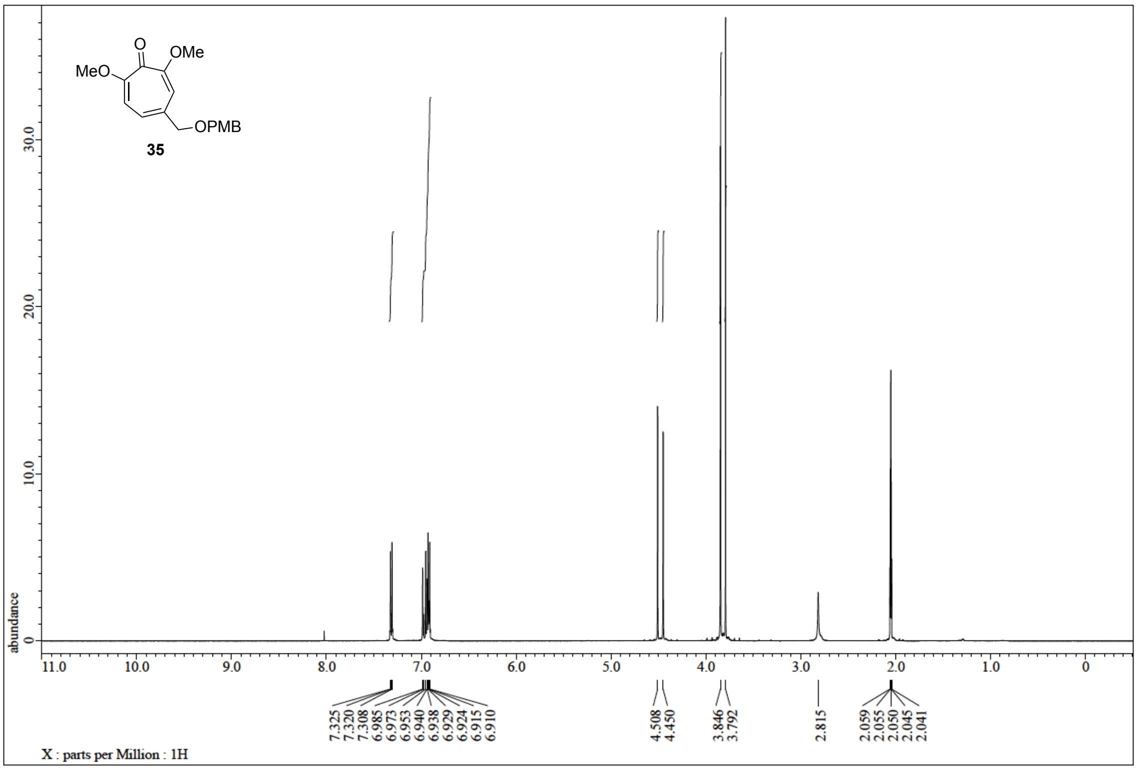

MeO
O
OMe
OPMB
35
abundance
X : parts per Million : 1H
7.325
7.320
7.308
6.985
6.973
6.953
6.940
6.938
6.929
6.924
6.915
6.910
4.508
4.450
3.846
3.792
2.815
2.059
2.055
2.050
2.045
2.041

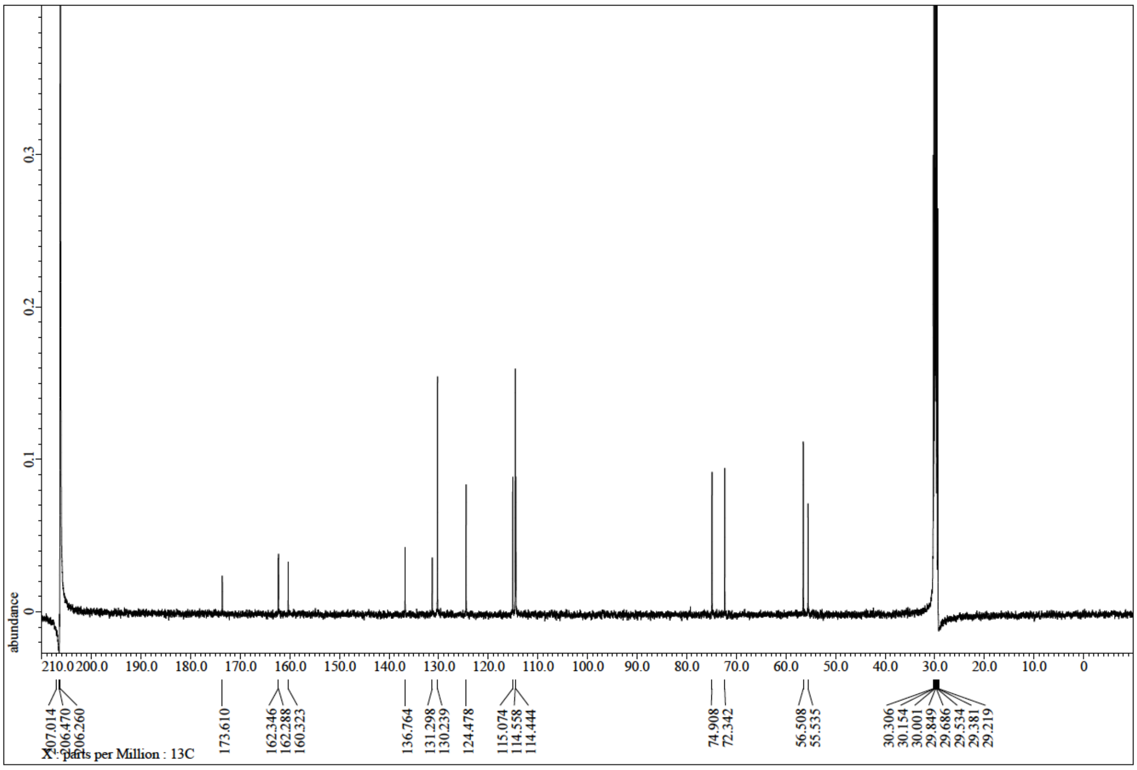

abundance
X : parts per Million : 13C
173.610
162.346
162.288
160.323
136.764
131.298
130.239
124.478
115.074
114.558
114.444
74.908
72.342
56.508
55.535
30.306
30.154
30.001
29.849
29.686
29.534
29.381
29.219

Alcohol **37**

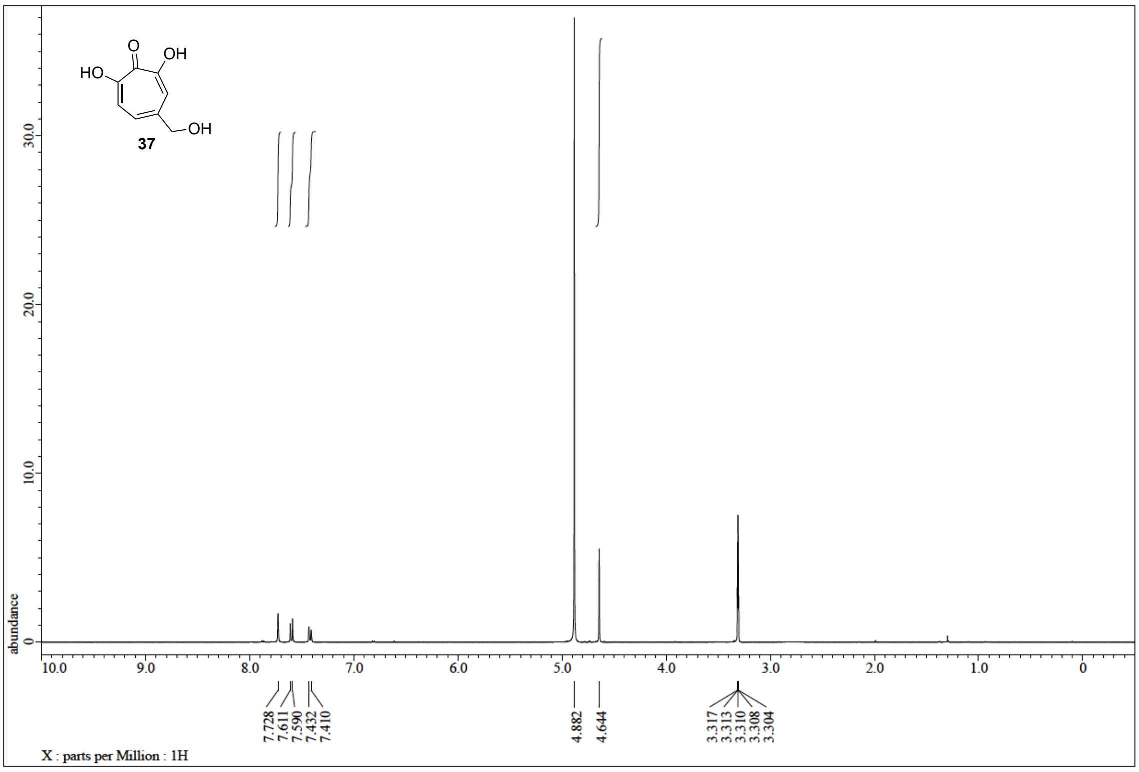

HO
O
OH
OH
37
abundance
7.728
7.611
7.590
7.432
7.410
4.882
4.644
3.317
3.313
3.310
3.308
3.304
X : parts per Million : 1H

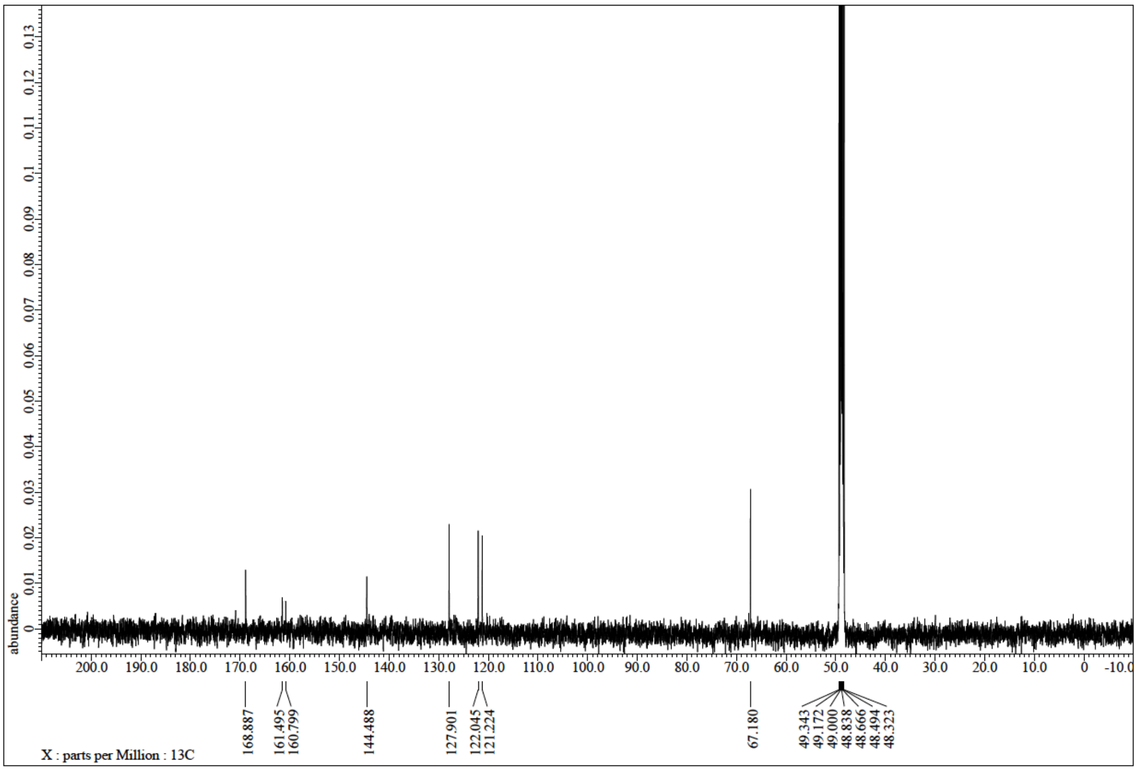

abundance
168.887
161.495
160.799
144.488
127.901
122.045
121.224
67.180
49.343
49.172
49.000
48.838
48.666
48.494
48.323
X : parts per Million : 13C

Methylether **38**

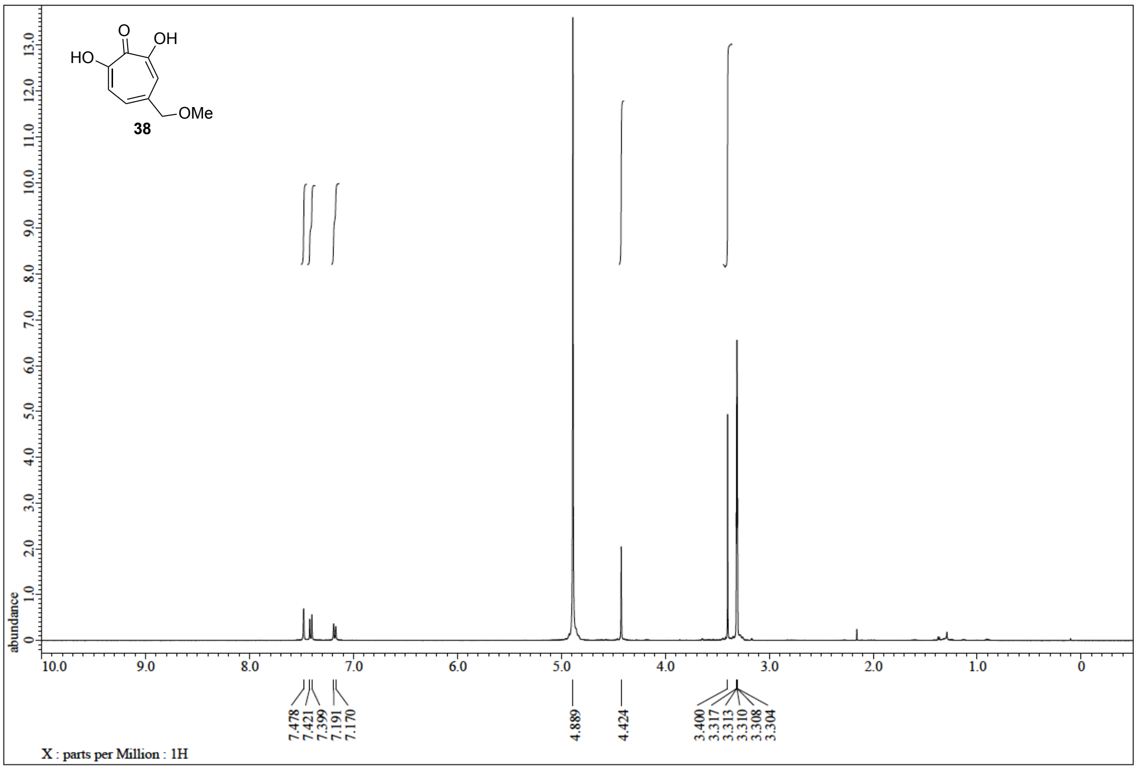

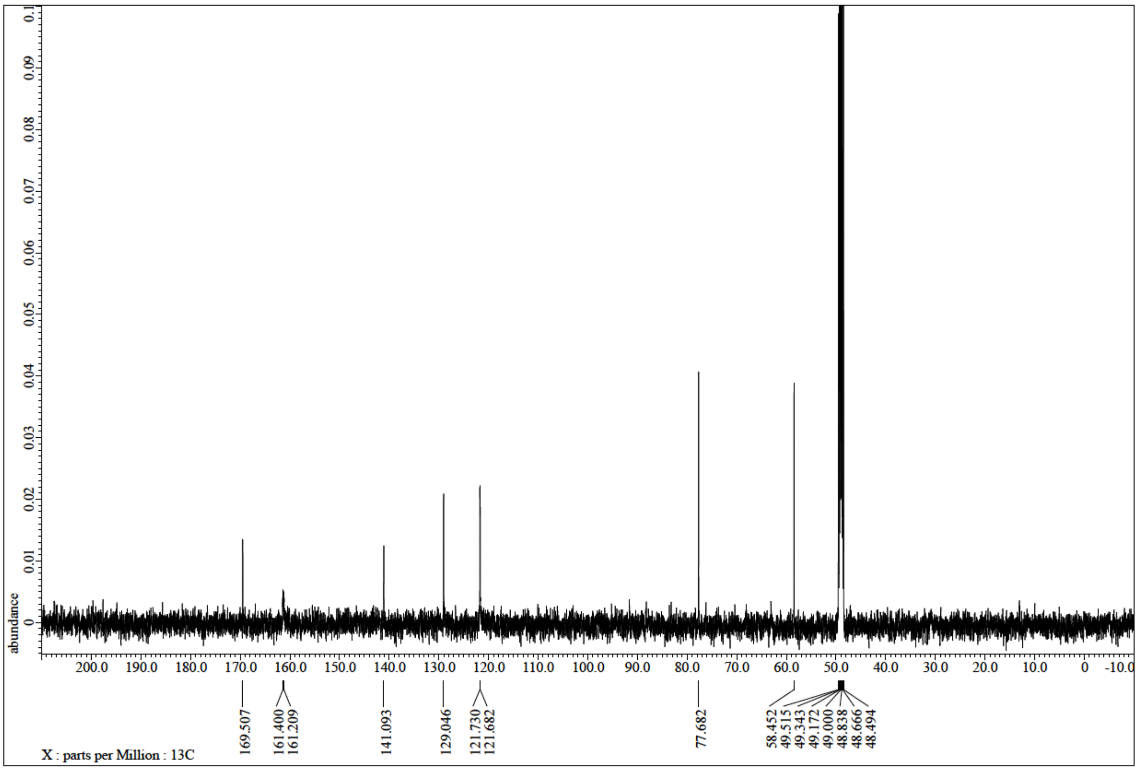

Aldehyde **39**

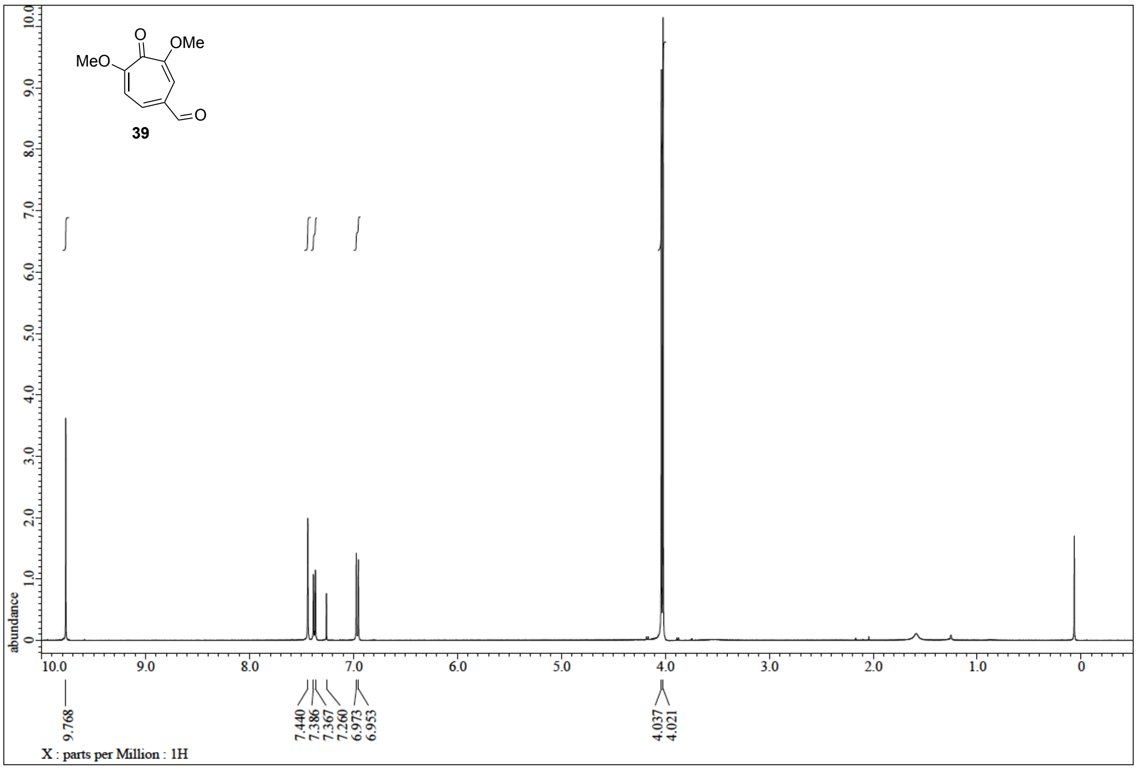

MeO
OMe
O
39
abundance
10.0
9.0
8.0
7.0
6.0
5.0
4.0
3.0
2.0
1.0
0
9.768
7.440
7.386
7.367
7.260
6.973
6.953
4.037
4.021
X : parts per Million : 1H

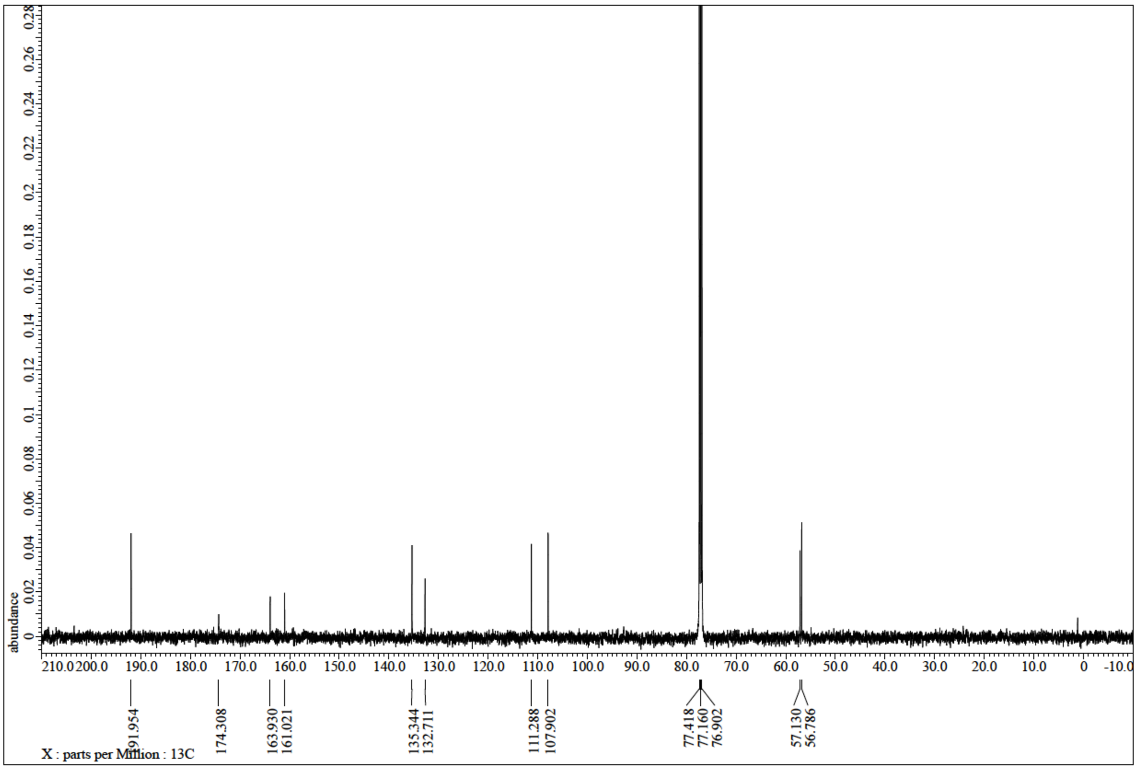

abundance
191.954
174.308
163.930
161.021
135.344
132.711
111.288
107.902
77.418
77.160
76.902
57.130
56.786
X : parts per Million : 13C

Alcohol **S1**

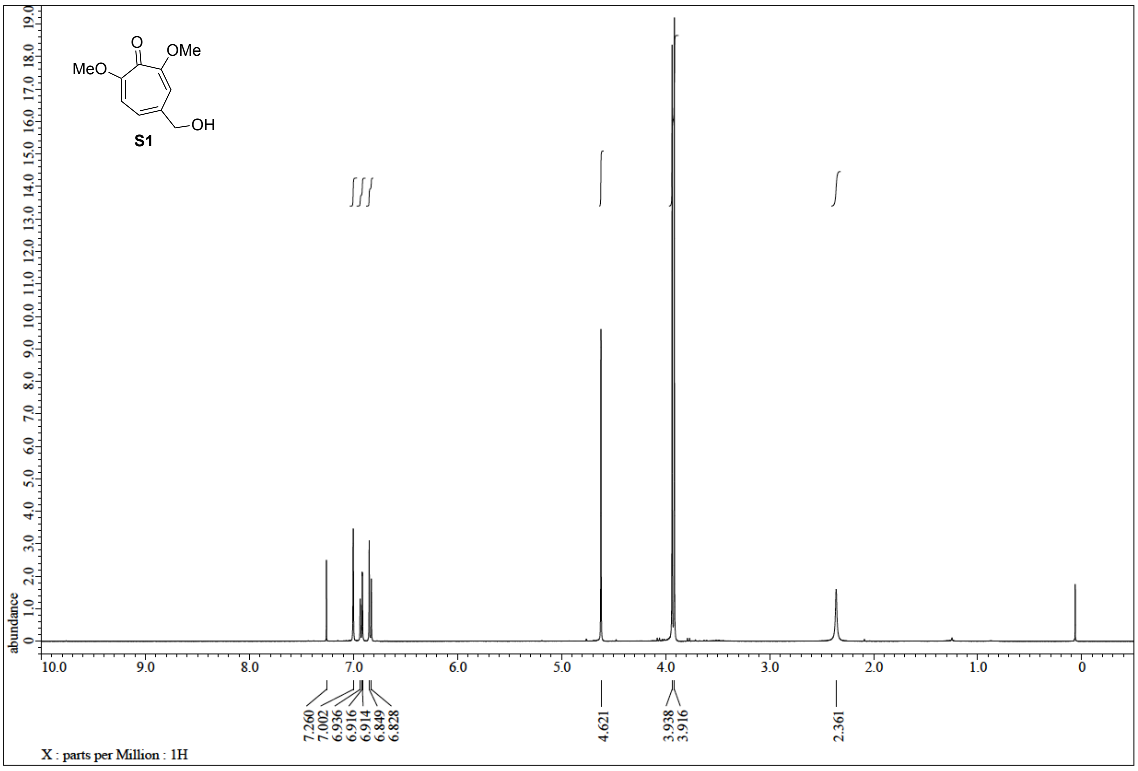

MeO
O
OMe
OH
S1
abundance
X : parts per Million : 1H
7.260
7.002
6.936
6.916
6.914
6.849
6.828
4.621
3.938
3.916
2.361

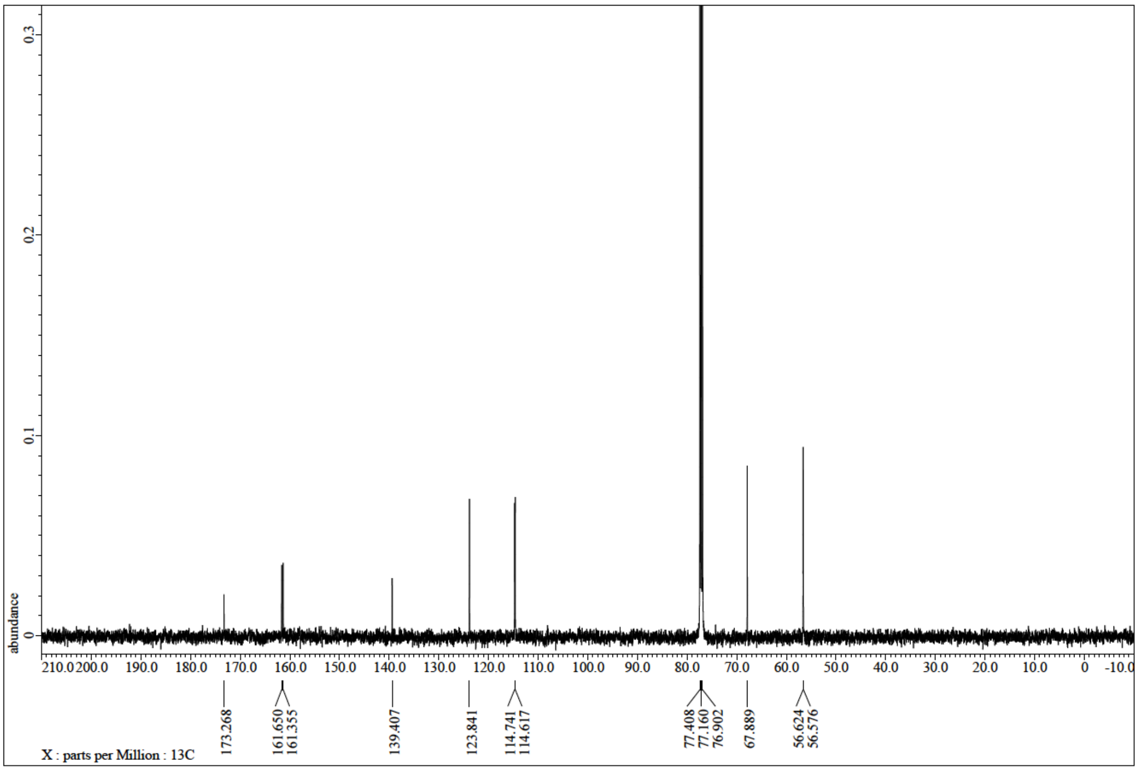

abundance
X : parts per Million : 13C
173.268
161.650
161.355
139.407
123.841
114.741
114.617
77.408
77.160
76.902
67.889
56.624
56.576

Iso-stipitatic acid (**40**)

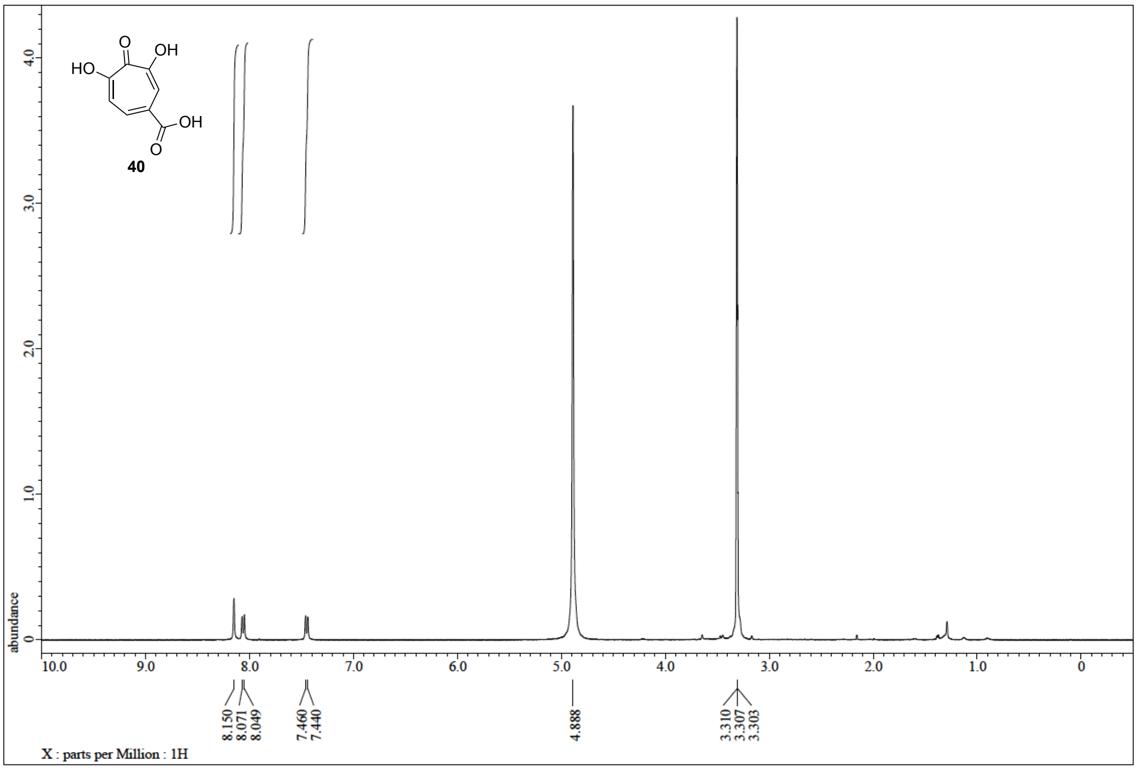

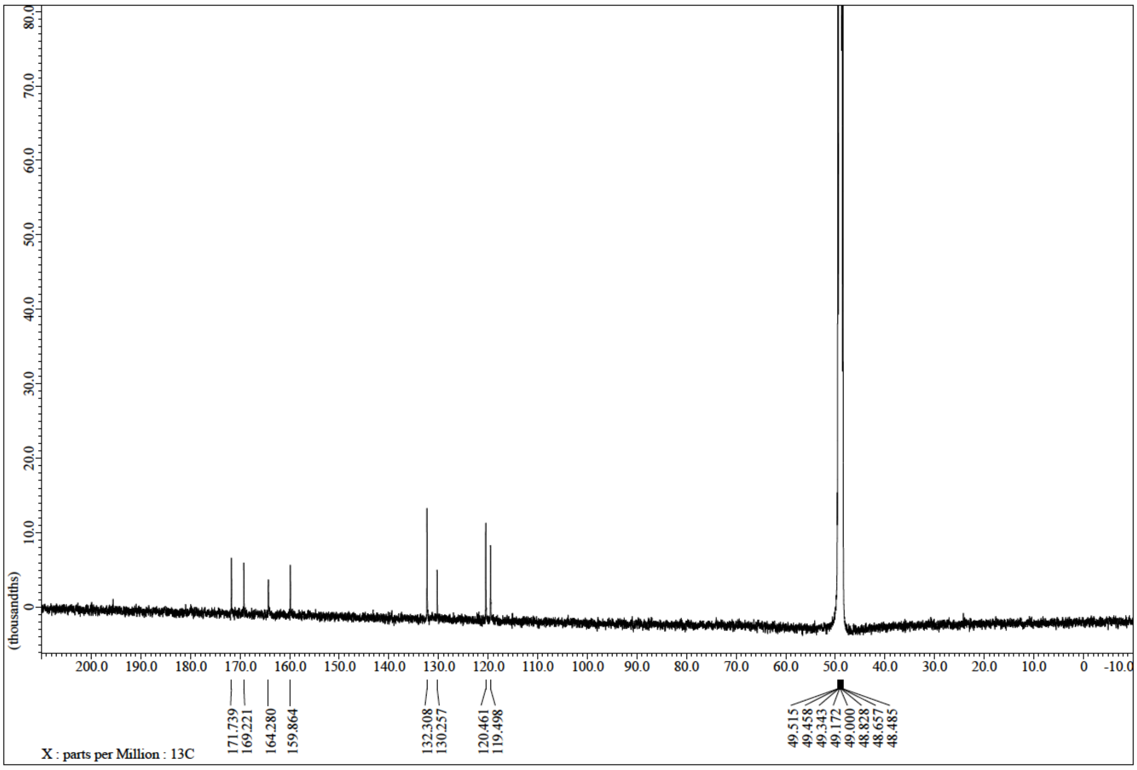

Alcohol **22**

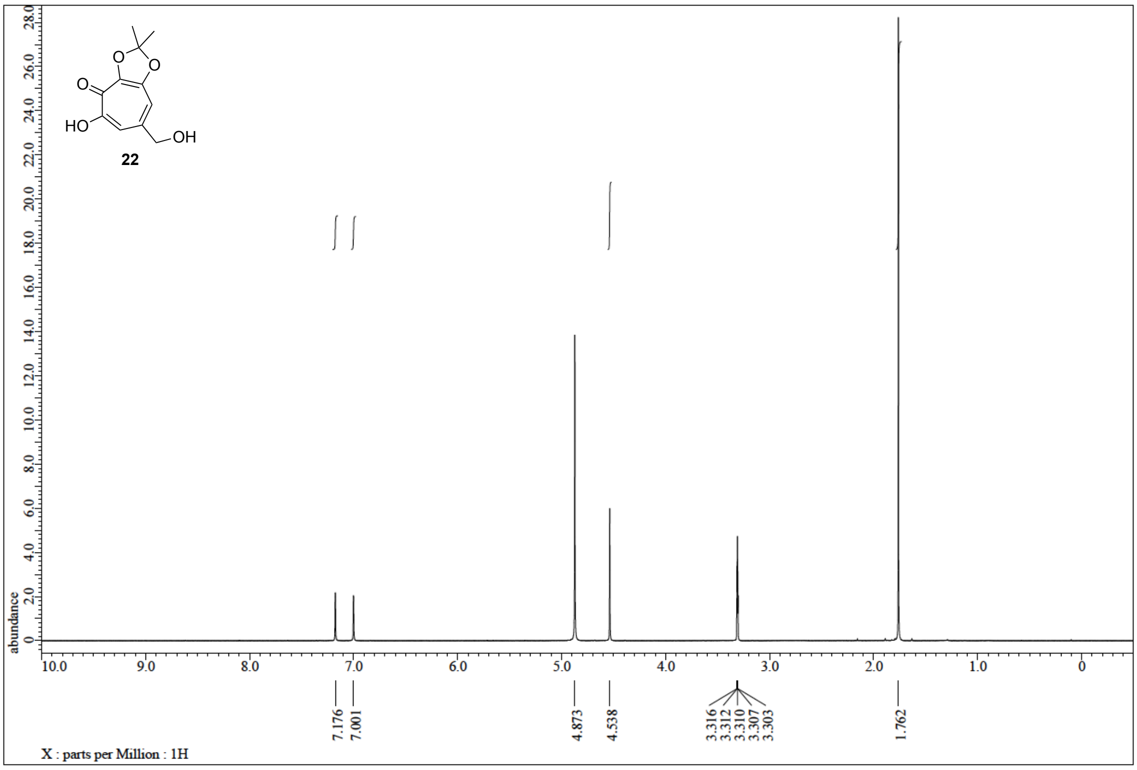

22
OH
HO
O
7.176
7.001
4.873
4.538
3.316
3.312
3.310
3.307
3.303
1.762
abundance
X : parts per Million : 1H

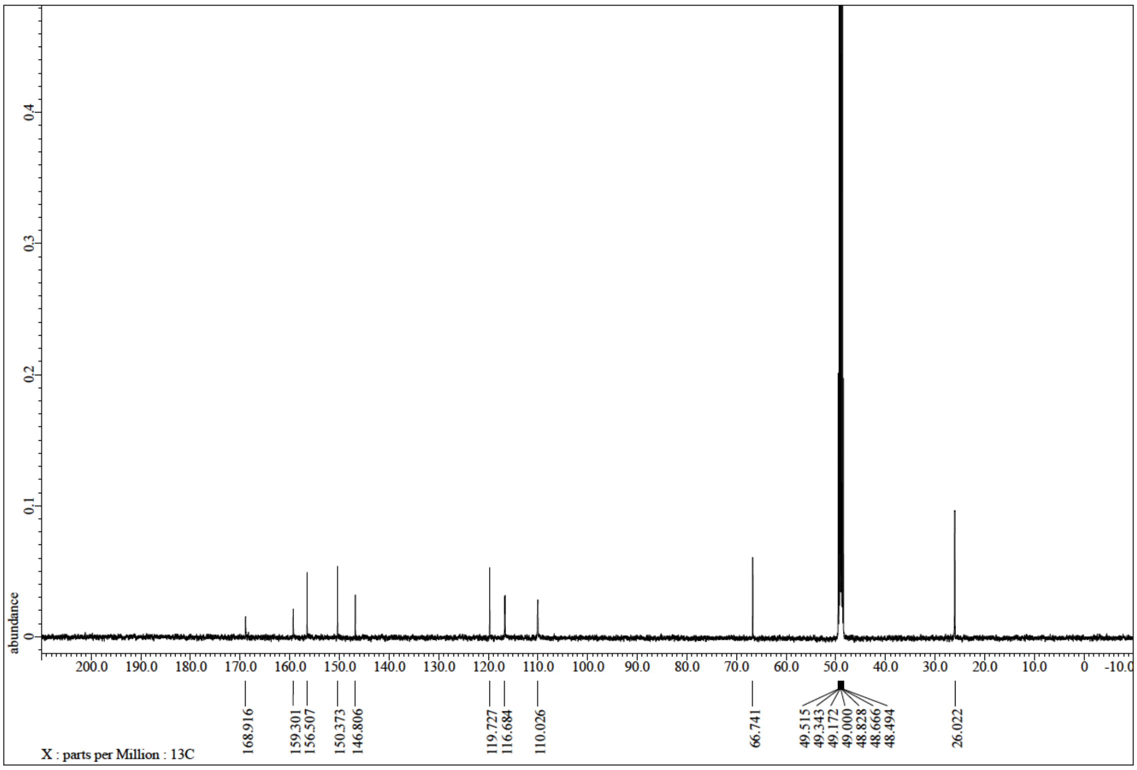

168.916
159.301
156.507
150.373
146.806
119.727
116.684
110.026
66.741
49.515
49.343
49.172
49.000
48.828
48.666
48.494
26.022
abundance
X : parts per Million : 13C

6,7-Dihydroxytropolone **42**

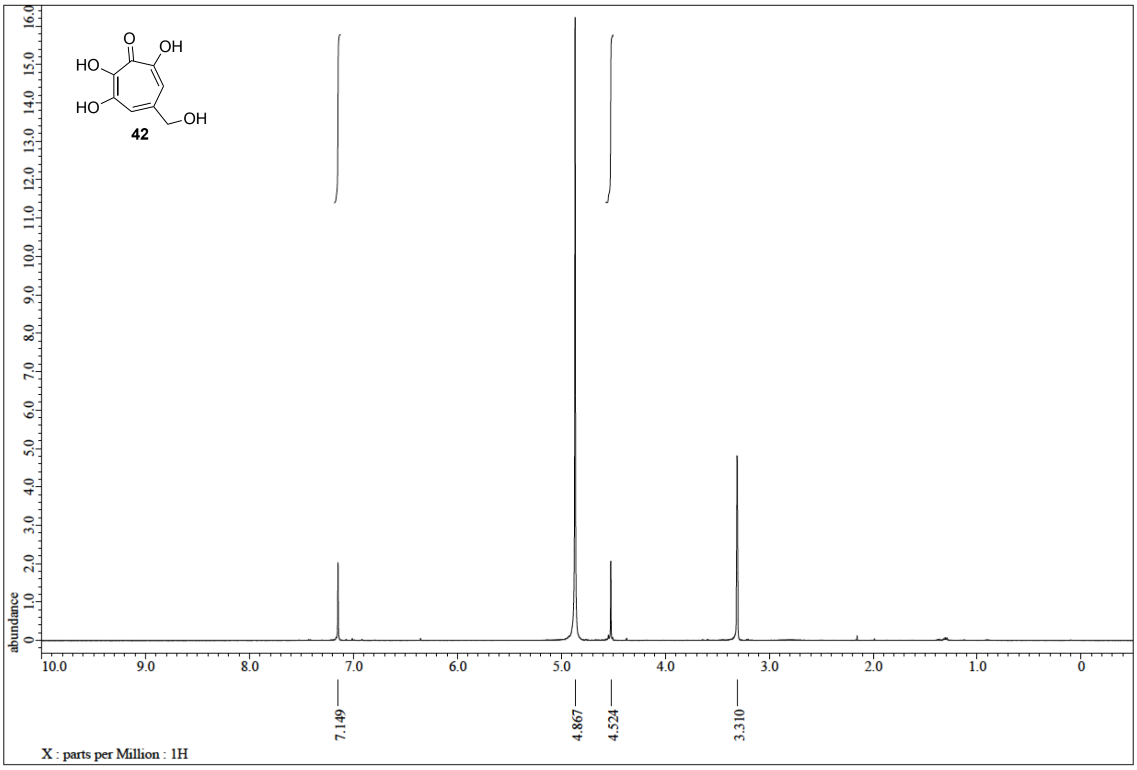

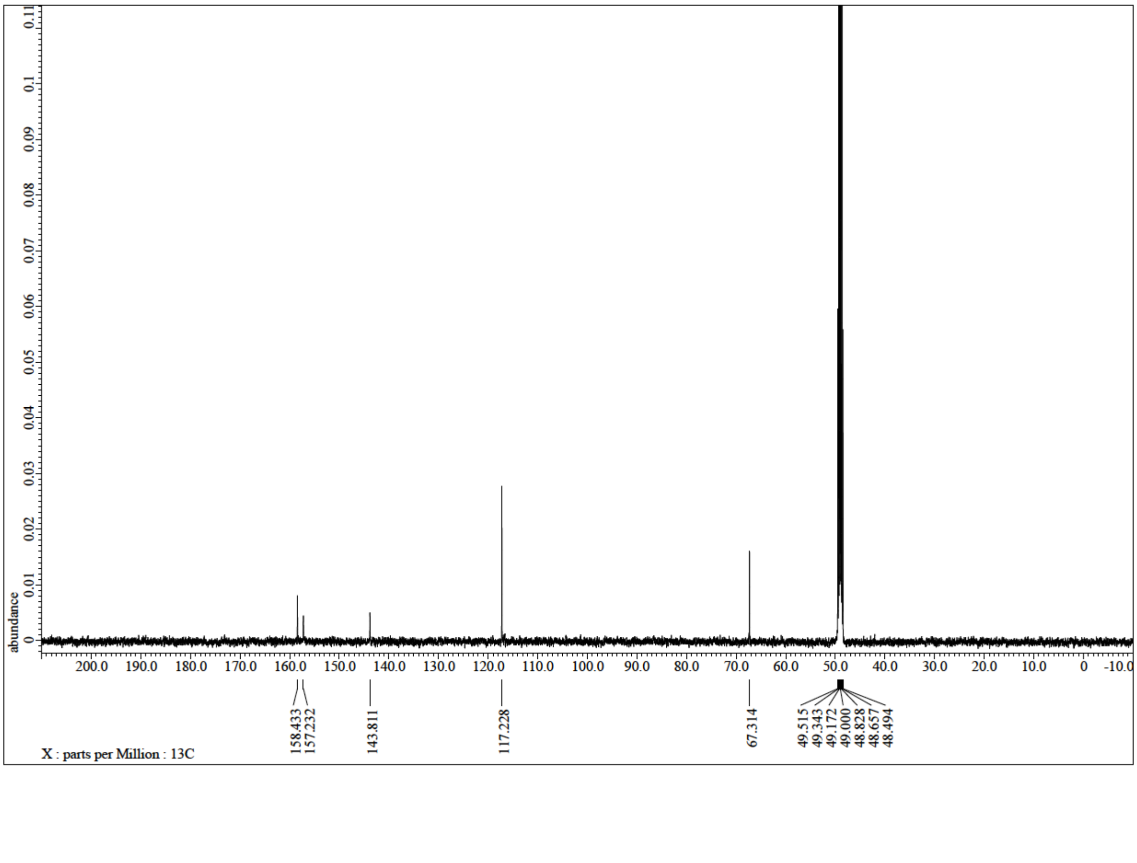

6,7-Dihydroxytropolone **43**

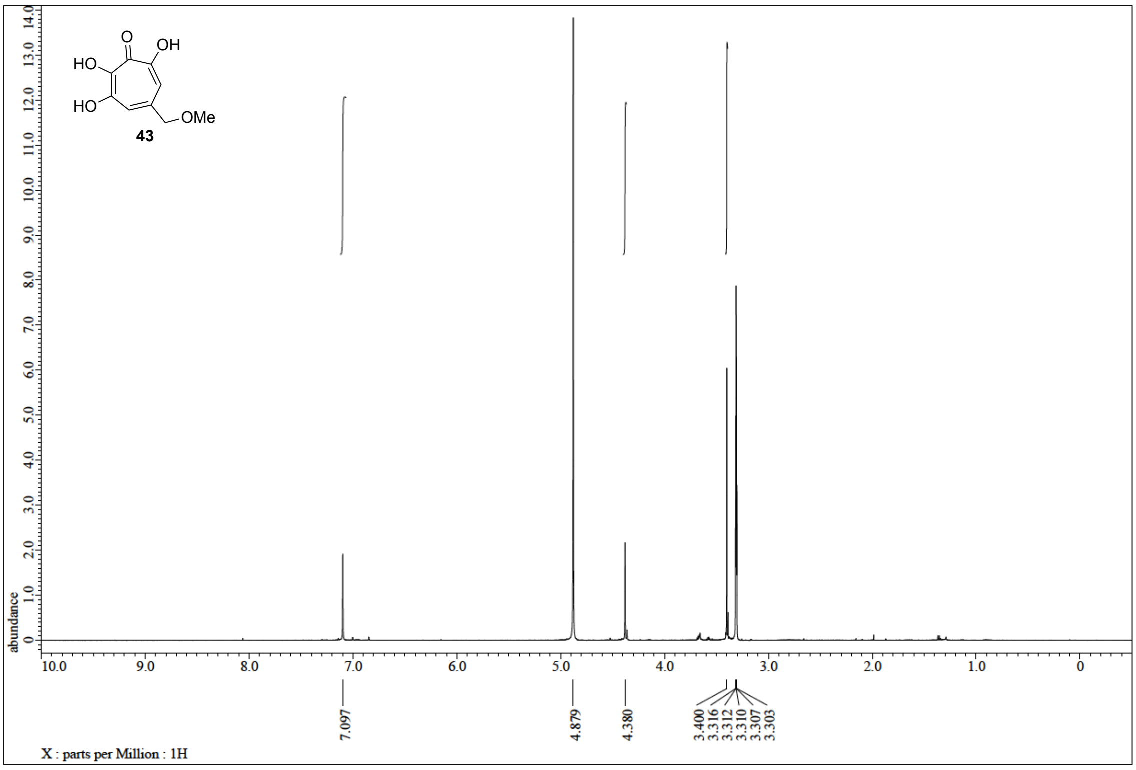

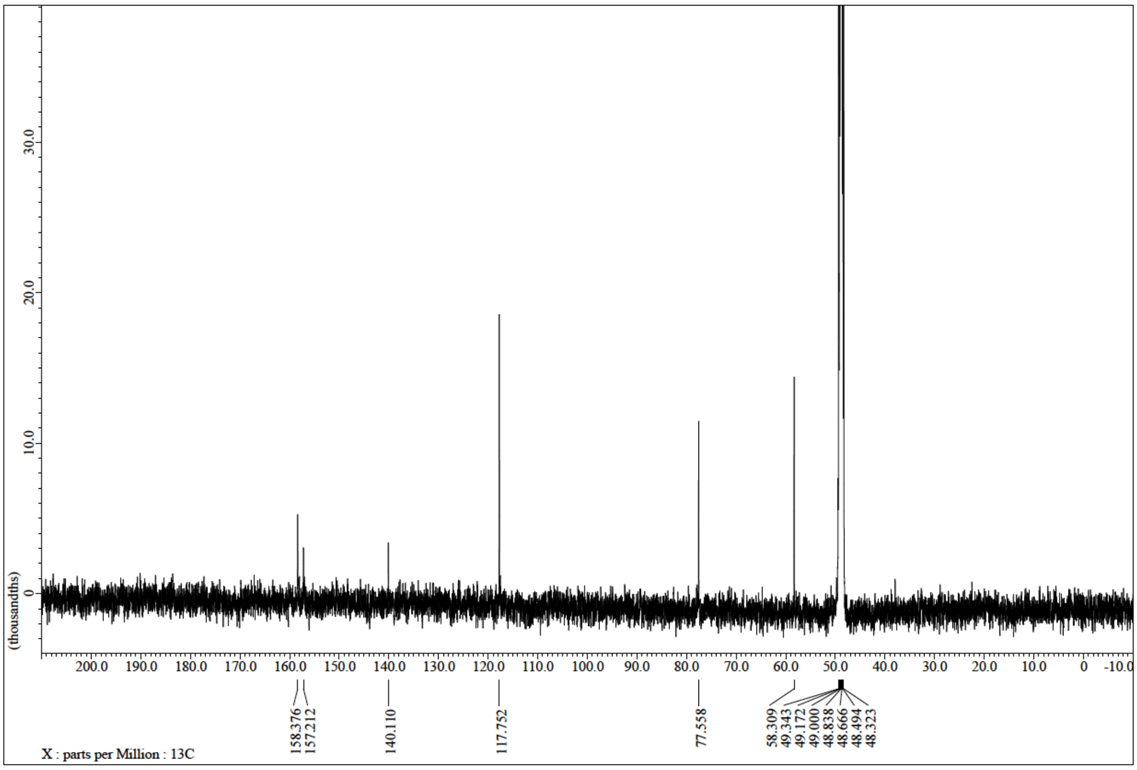

Azide **44**

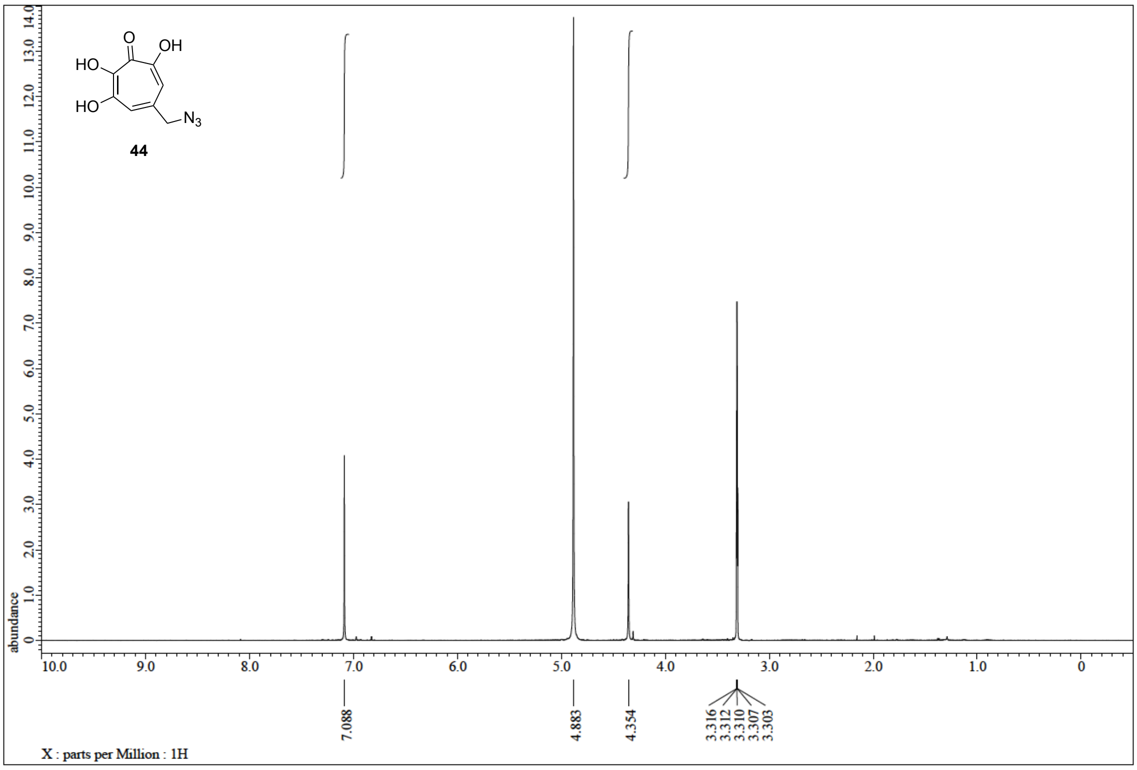

O
OH
HO
HO
N3
44
abundance
10.0 9.0 8.0 7.0 6.0 5.0 4.0 3.0 2.0 1.0 0
7.088
4.883
4.354
3.316
3.312
3.310
3.307
3.303
X : parts per Million : 1H

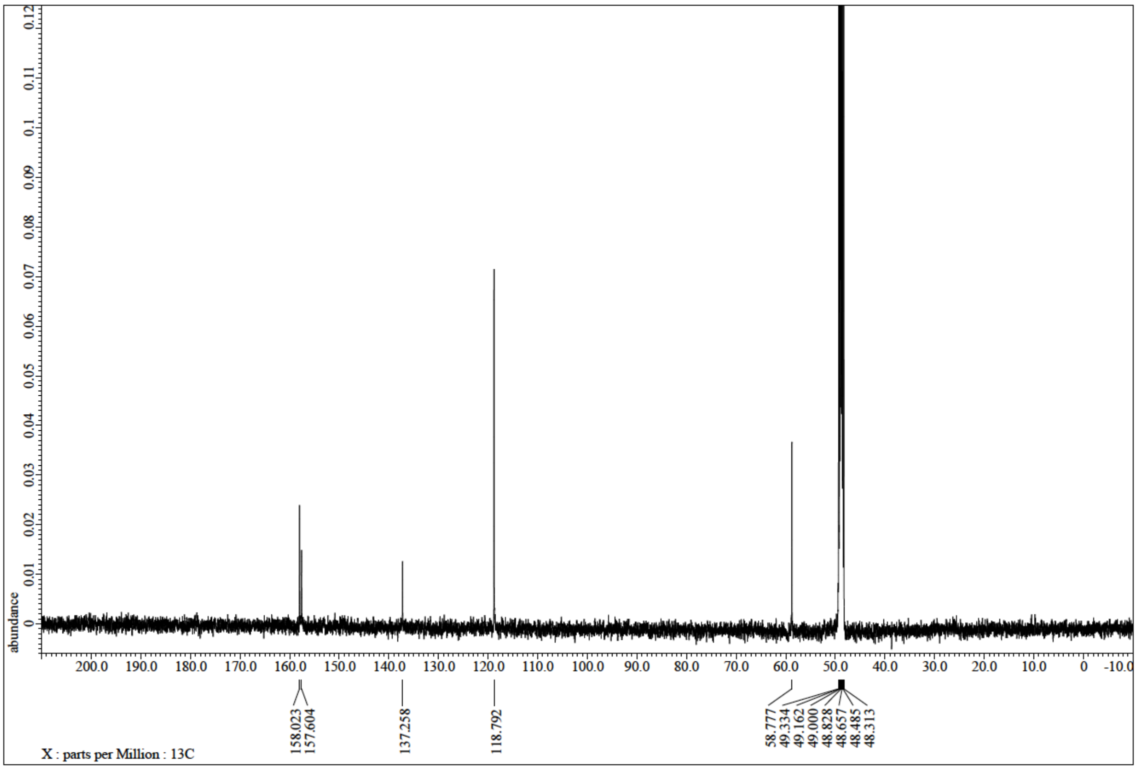

abundance
200.0 190.0 180.0 170.0 160.0 150.0 140.0 130.0 120.0 110.0 100.0 90.0 80.0 70.0 60.0 50.0 40.0 30.0 20.0 10.0 0 -10.0
158.023
157.604
137.258
118.792
58.777
49.334
49.162
49.000
48.828
48.657
48.485
48.313
X : parts per Million : 13C
